# Genomic and Functional Characterization of the Endophytic *Bacillus siamensis* Strain BACIII with Plant Growth-Promoting and Antifungal Activity

**DOI:** 10.3390/microorganisms14071569

**Published:** 2026-07-17

**Authors:** Jefferson Brendon Almeida dos Reis, Sofia Coradini Schirmer, Maria Regina Silveira Sartori da Silva, Andrei Stecca Steindorff, Patrícia Cardoso Cortelo, Georgios Joannis Pappas, Helson Mario Martins do Vale

**Affiliations:** 1 Institute of Biological Sciences, University of Brasilia, Brasília 70910-900, DF, Brazil; jeffersonalmeidareis@gmail.com (J.B.A.d.R.); sofiaschirmer@gmail.com (S.C.S.); pattycar8@gmail.com (P.C.C.);; 2 Lawrence Berkeley National Laboratory, US DOE Joint Genome Institute, Berkeley, CA 94720, USA

**Keywords:** *Bacillus siamensis*, biological control, secondary metabolites, soybean, sunn hemp

## Abstract

Endophytic bacteria of the *Bacillus subtilis* species complex are known for plant growth promotion and antifungal activity, although strain-specific traits remain poorly understood. This study characterized the *Bacillus siamensis* strain BACIII, isolated from asymptomatic soybean roots in a charcoal rot-affected area, using whole-genome analysis and phenotypic assays. Genome sequencing identified BACIII as *Bacillus siamensis* and revealed a metabolically versatile genome containing twelve biosynthetic gene clusters linked to antimicrobial compounds such as difficidin, fengycin, and surfactin. Additionally, genomic islands associated with mobile elements, regulation, and stress response suggest adaptive potential. Inoculation with the BACIII strain significantly accelerated germination and increased early growth, biomass accumulation, and chlorophyll content in soybean and sunn hemp (*p* < 0.05), whereas no significant effects were observed in cotton or sunflower, indicating host-dependent responses. In vitro assays demonstrated consistent inhibition of several phytopathogenic fungi, including *Sclerotinia sclerotiorum, Fusarium* spp., and *Macrophomina phaseolina*, with variable intensity. Overall, BACIII combines host-specific plant growth promotion with broad antifungal activity, supporting its potential for biological control in sustainable agriculture.

## 1. Introduction

The increasing pressure to reconcile high agricultural productivity with environmental sustainability has driven the development of alternative strategies aimed at reducing the intensive use of synthetic fertilizers and pesticides [1,2]. Microorganisms capable of establishing symbiotic associations with plants, particularly mutualistic interactions in which both partners benefit, have gained prominence due to their ability to promote plant development (see reviews by Liu-Xu et al., 2024 [3] and Sahoo et al., 2025 [4]). Among these microorganisms, rhizobacteria classified as plant growth-promoting rhizobacteria (PGPR) stand out, as they are capable of promoting plant growth under different environmental conditions [5], including both abiotic stress [6] and biotic stress [7,8]. The growth-promoting effects associated with PGPR mainly result from direct and indirect interaction mechanisms, which can lead to changes in the morphology and physiology of the roots of inoculated plants [9,10], competition with phytopathogenic microorganisms [8], nutrient solubilization [11], and the production of plant hormones or secondary metabolites [12,13]. As a consequence of these interactions, plants tend to exhibit improved root system development, with increased biomass and root hairs [9,10], enhanced shoot growth [8,14], as well as greater productivity and tolerance to stresses such as diseases [8], salinity [15], and water deficit [16]. Among microorganisms classified as PGPR, bacteria of the genus *Bacillus* have received special attention due to their metabolic and ecological versatility [6].

*Bacillus* is a genus of Gram-positive bacteria belonging to the phylum Bacillota (Firmicutes), characterized by their ability to form endospores and by their ubiquitous distribution across natural environments. Species of *Bacillus* can colonize soils [17,18], aquatic environments [19], and extreme habitats [20,21,22], as well as establish symbiotic associations with plants [23,24]. In general, *Bacillus* spp. are considered metabolically versatile bacteria, capable of colonizing a wide range of substrates and environments under different regimes of resource availability [25] and variations in pH [26,27], osmolarity [20,21], and temperature [22,28]. In addition, different species within this genus can establish symbiotic and mutualistic ecological interactions with plants. A notable example includes species belonging to the so-called *B. amyloliquefaciens* operational group, which is part of the *B. subtilis* complex [29,30]. Species such as *B. velezensis* and *B. siamensis* have been widely reported as plant growth promoters, acting through multiple mechanisms that include the inhibition of phytopathogens, nutrient mobilization, and the production of bioactive metabolites [31,32,33].

Despite the large number of strains described with PGPR potential, the variability in performance observed under real cultivation conditions highlights the need for a deeper understanding of the genetic determinants underlying these phenotypes. Given this scenario, genomics has emerged as an indispensable approach for the characterization of promising *Bacillus* strains. Whole-genome sequencing and annotation enable the identification of genes and biosynthetic gene clusters (BGCs) associated with the production of bioactive compounds, plant colonization, and adaptation to edaphic environments [34,35]. Thus, integrating genomic data with phenotypic assays allows for more robust correlations between the plant growth-promoting potential of this bacterial genus and its functional traits. However, despite the increasing number of studies involving *Bacillus* spp. [31,32,33], important gaps remain regarding its agricultural application, particularly concerning the consistency of plant growth-promoting effects across different host species and the extent to which genomic traits are linked to observed phenotypic responses under biological assays. In many cases, strains described as PGPR have been evaluated only in single-host systems, limiting broader inferences about host-dependent interactions and functional performance.

In this context, we conducted an integrated investigation of the plant growth-promoting potential and antagonistic capacity of a *Bacillus siamensis* strain (BACIII) against phytopathogenic fungi. The BACIII strain was isolated as an endophyte from soybean roots collected in an agricultural area with a history of recurrent charcoal rot. We hypothesized that this strain possesses a set of functional attributes that enable it to both stimulate germination and promote the initial and vegetative growth of agricultural plants belonging to different botanical families. To test this hypothesis, we evaluated the effects of inoculation on germination parameters and on the morphophysiological development of the plants. Additionally, we investigated the ability of the BACIII strain to inhibit phytopathogenic fungi across different taxonomic classes, recognizing that distinct patterns of sensitivity may reflect variation in the underlying antagonistic mechanisms. In parallel, we performed a complete genomic characterization of the BACIII strain, with emphasis on the prediction of BGCs and functional genes associated with secondary metabolite biosynthesis, plant colonization, and modulation of microorganism–plant interactions.

## 2. Materials and Methods

### 2.1. Bacterial and Fungal Strains and Seeds

The bacterial strain was isolated from soybean (*Glycine max*) roots collected in an area affected by charcoal rot. The strain was isolated after soybean roots had been subjected to a surface sterilization procedure [36]. The final rinse water was collected to confirm the effectiveness of surface sterilization. Root fragments (~1 cm) were placed on potato dextrose agar (PDA) and incubated in a B.O.D. chamber (Biochemical Oxygen Demand) at 25 ± 2 °C for 7 days. After incubation, the isolate was purified on PDA and preserved in the Culture Collection of the University of Brasília using the Castellani method and 10% (v/v) glycerol stocks. The fungal isolates used in this study were obtained from the fungal culture collection of the University of Brasília. Seeds of four cultivated species were employed in the germination and growth: soybean (*Glycine max*, cv. Brasmax Olimpo IPRO 80I82RSF), sunn hemp (*Crotalaria juncea*, cv. C52333-C), cotton (*Gossypium hirsutum*, cv. FM 985 GLTP), and sunflower (*Helianthus annuus*, cv. Hélio 251). provided by VL Agronegócios—Laboratório de Análises Agronômicas (Catalão, Goiás, Brazil).

### 2.2. DNA Extraction

For DNA extraction, the bacterial strain was first grown on PDA medium for 48 h at 25 °C. A bacterial inoculum at a concentration of 1 × 10^6^ CFU·mL^−1^ was then transferred to 250 mL of potato dextrose broth and incubated under the same conditions. After 48 h, the culture was centrifuged at 9000 rpm for 10 min in 50 mL Falcon tubes. The supernatant was discarded, and the pellet containing the bacterial cells was collected. Genomic DNA was extracted following dos Reis et al. [36], with modifications. Briefly, the bacterial pellet was frozen in liquid nitrogen and ground with a mortar and pestle until a homogeneous white powder was obtained. Approximately 400 mg of this powder was added to 1000 µL of CTAB buffer (7–10% CTAB, 100 mM Tris-HCl, 20 mM EDTA, pH 8.0, 1.4 mM NaCl) plus 150 mg of polyvinylpyrrolidone 40. The microtubes were incubated at 65 °C in a dry bath for 15 min, with vigorous mixing every five minutes. After incubation, 600 µL of chloroform:isoamyl alcohol (24:1) solution was added to each tube, and the mixture was gently inverted for ten minutes. Tubes were then centrifuged at 14,000 rpm for ten minutes at 10 °C, and the aqueous phase was transferred to new 1.5 mL microtubes containing 55 µL of CTAB (7%). An additional 600 µL of chloroform:isoamyl alcohol (24:1) was added, followed by gentle inversion for 10 min and centrifugation under the same conditions. This step was repeated until no interface between the aqueous and organic phases remained.

Approximately 800 µL of the aqueous phase was transferred into new tubes containing 800 µL of chilled propanol, mixed by gentle inversion for five minutes, and incubated at −20 °C for two hours. The tubes were centrifuged at 14,000 rpm to precipitate the DNA. The supernatant was discarded, and the pellet was washed once with 700 µL of 70% ethanol and twice with 700 µL of absolute ethanol (99.8%), with centrifugation at 14,000 rpm for five minutes between washes. The pellet was air-dried at room temperature for one hour and rehydrated in 100 µL of TE (10 mM Tris-HCl, 1 mM EDTA, pH 8.0). DNA integrity was checked on 1% agarose gels stained with GelRed^®^ and visualized under UV light after electrophoresis. DNA purity was assessed using a Nanodrop, considering acceptable absorbance ratios of 1.8–2.0 (A260/280) and 2.0–2.2 (A260/230).

### 2.3. Genome Sequencing, Assembly, and Annotation

The DNA sequencing library was prepared using the Rapid Barcoding 96 kit (SQK-RBK114.96) from Oxford Nanopore Technologies (ONT), following the manufacturer’s instructions. The library was loaded onto an R10.4.1 flow cell and sequenced on a MinION Mk1B platform. Basecalling and demultiplexing were performed using Dorado v.0.7.2 with the super-accurate model (dna_r10.4.1_e8.2_400bps_sup@v5.0.0).

Following sequencing, raw reads were processed using the pipelines implemented by de Almeida et al. [37]. These procedures consist of three main stages: quality control (ngs-preprocess v2.7.1; https://github.com/fmalmeida/ngs-preprocess; accessed on 12 February 2026), de novo genome assembly (MpGAP v3.2.3; https://github.com/fmalmeida/MpGAP; accessed on 12 February 2026), and bacterial genome annotation (bacannot v3.4.4; https://github.com/fmalmeida/bacannot, accessed on 12 February 2026). Within MpGAP, several assembly programs are systematically evaluated, and the assembler providing the highest contiguity is selected; in this analysis, Canu v2.2 [38] yielded the optimal assembly. The final assembly consensus was obtained after error correction using Medaka v1.5.0 (https://github.com/nanoporetech/medaka; accessed on 12 February 2026).

The resulting assemblies were annotated with Prokka v1.14.6 [39]. Circular genome visualization was generated using Bakta Web [40,41]. Biosynthetic gene cluster predictions were performed using antiSMASH v8.0.4 with default settings and “relaxed” mode enabled [42]. Effector protein prediction was performed using SignalP v6.0 [43]. Genomic islands were predicted using the IslandViewer 4 platform [44]. Functional classification and pathway inference were conducted using BlastKOALA, which assigns KEGG Orthology (KO) identifiers and enables the identification of metabolic pathways and functional modules encoded in the genome [45].

Plant-associated functional traits were predicted using the PLabASE platform (v24.04.3) [45]. Annotation of plant–bacteria interaction factors (PIFARs) was conducted through BLASTP and HMMER searches against a curated database of PIFAR proteins in strict mode [46,47]. In parallel, plant growth-promoting traits (PGPTs) were identified based on protein sequences using BLASTP and HMMER (strict mode), allowing the classification of genes into functional categories associated with plant growth promotion [46,47,48].

### 2.4. Phylogenetic Inferences and Pairwise Comparison of Genomic Sequences

For phylogenetic inference, we first used BLASTn [49] to perform a similarity search against the non-redundant NCBI database, using the 16S rRNA sequence from the genome of strain BACIII as the query. The result showed 100% identity and coverage with *Bacillus velezensis* (CP089310.1; CP128503.1; CP152037.1). This species belongs to the “Operational Group *B. amyloliquefaciens*” within the *B. subtilis* complex [29]. Because there is no established taxonomic consensus for genome-level comparisons within this complex [50,51,52], we selected genomes available in the JSpeciesWS search [53], including type strains, which were also accessible through the Genbank platform. The genomes used represent *B. amyloliquefaciens*, *B. siamensis* and *B. velezensis* (complete list: GCF_001461825.1, GCF_001267695.1, GCF_001461835.1, GCF_001461845.1, GCF_001440465.1, GCF_001469675.1, GCF_000769555.1, GCF_000319475.1, GCF_000283695.1, GCF_000015785.1, GCF_000833005.1, GCF_000973485.1, GCF_000960265.2, GCF_000740715.1, GCF_000262045.1, GCF_001662915.1, GCF_000196735.1, GCF_965136255.1, GCF_050473685.1, GCF_000750045.1, GCF_001278635.1, GCF_001286945.1, GCF_001286965.1, GCF_000204275.1, GCF_000508265.1, GCF_000835145.1 and GCF_000242855.2). Comparative analyses were conducted using ANIb (Average Nucleotide Identity based on BLAST), ANIm (Average Nucleotide Identity calculated with MUMmer) and Tetranucleotide Frequency Correlation Analysis, both performed on JSpeciesWS [53]. The 16S rRNA gene sequence has been deposited in GenBank under accession number PP732323.1. The Whole Genome Shotgun project has been deposited in DDBJ/ENA/GenBank under accession number JBTIYL000000000. The genome assembly described in this study corresponds to version JBTIYL010000000.

We also applied the Genome BLAST Distance Phylogeny (GBDP) method using the automated Type Strain Genome Server (TYGS) [54]. The analysis incorporated recent methodological updates and resources described in Meier-Kolthoff et al. [55] and Freese et al. [56]. Information on nomenclature, synonymy, and taxonomic literature was retrieved from the complementary TYGS database, the List of Prokaryotic names with Standing in Nomenclature (LPSN; available at https://lpsn.dsmz.de; accessed on 12 February 2026). The TYGS analysis followed the standard workflow described for the platform. The identification of the closest type-strain genomes was performed in two complementary steps. First, the genome of strain BACIII, along with the genomes used in the JSpecies analyses, was compared against all type-strain genomes available in the TYGS database using the MASH algorithm, which provides a rapid approximation of intergenomic relatedness [57]. For each genome, the ten type strains with the smallest MASH distances were selected. In the second step, an additional set of type strains was identified using 16S rDNA sequences. These sequences were extracted with RNAmmer [58] and subjected to BLAST searches [59] against the 16S rDNA sequences of the 23,877 type strains currently available in the TYGS database. Based on bit score ranking, the 50 closest type strains were retrieved and used to calculate precise intergenomic distances through the Genome BLAST Distance Phylogeny approach under the coverage algorithm and the d5 distance formula [60]. From these results, the ten closest type-strain genomes were selected.

For the phylogenomic inference, all pairwise comparisons within the final genome set were computed using GBDP with the trimming algorithm and the d5 distance formula. One hundred distance replicates were generated for each comparison. Digital DDH values and confidence intervals were obtained with the recommended settings of GGDC 4.0 [55,60]. The resulting distance matrices were used to infer a balanced minimum-evolution tree in FASTME 2.1.6.1, including SPR post-processing [61]. Branch support was estimated from one hundred pseudo-bootstrap replicates. Trees were midpoint-rooted [62] and visualized in PhyD3 [63]. Species delimitation followed the type-based clustering approach with a 70% dDDH radius around each of the 21 type strains, as described previously [54]. Subspecies-level clusters were defined using the 79% dDDH threshold [64].

### 2.5. Genomic Comparison

For the genomic comparison, we selected four type-strain genomes, including the species reference genome from GenBank, based on our ANIm, TETRA, and TYGS results. Selection criteria focused on high-quality data: we prioritized sequencing completeness, a contig count of fewer than 3, and genomes derived from isolated strains rather than metagenomes. The selected accessions were GCF_002850535.1, GCF_026014025.1, GCF_024453785.1, and GCF_016313165.1. We used the OrthoVenn3 web service to identify and annotate orthologous protein-coding gene clusters and to infer phylogenetic relationships [65]. Whole-genome alignments were performed using syri v.1.7.1 [66], and the resulting alignments visualized using plotsr v.1.1.0 [67].

### 2.6. Seed Germination Test

Germination assays were conducted following the methodology described by Pérez-García et al. [68] and Pappalettere et al. [69], with minor adaptations. The *Bacillus* strain BACIII was previously grown in YPD broth at 24 °C for 72 h without shaking. After growth, the culture was centrifuged at 9000 rpm for 10 min to pellet the bacterial cells. The bacterial pellet was rinsed twice with sterile distilled water and subsequently suspended in a 2% sucrose solution. The cell density was then adjusted to approximately 1 × 10^6^ CFU·mL^−1^, yielding the final inoculum suspension. For each species, 2000 seeds were used, with 1000 treated with the bacterial suspension and 1000 used as the negative control. Surface disinfection followed the protocol of dos Reis et al. [70]: immersing the seeds in 70% ethanol for 2 min, followed by sodium hypochlorite for 2 min, and then rinsing 5 times with sterile distilled water. The water from the final wash was collected and inoculated onto PDA plates to verify the effectiveness of the surface disinfection procedure. The seeds were dried in a laminar flow chamber and immersed in the bacterial suspension for 20 min. The negative control consisted of treatment with a 2% sucrose solution without bacteria. The seeds were then placed in Gerboxes containing two sheets of paper towel moistened with 15 mL of autoclaved distilled water. Each sample unit consisted of 100 seeds, with 10 replicates per treatment for each species (*n* = 10), except for sunflower, for which each Gerbox contained 70 seeds, totaling 14 replicates per treatment (*n* = 14; 980 seeds). The seeds were incubated in a B.O.D. chamber at 24 °C in the dark for 7 days.

Germination was recorded daily until the seventh day, and the Germination Speed Index (GSI), Germination Percentage (%) (GP%), and Seed Vigor (%) (SV%) were determined [68,71]. The GSI reflected the germination rate over time, while GP represented the total seed viability at the end of the experiment. Seed vigor was assessed on the fourth day, based on the cumulative percentage of germinated seeds at that point. At the end of the experiment, seedlings were collected for dry weight determination, used as an indicator of biomass accumulation and biostimulatory effect. The assessment considered seedling development: when more than 70% of germinated seeds produced complete seedlings, dry weight was determined for the whole seedling (hypocotyl, radicle, and cotyledons); when fewer than 70% formed complete seedlings, only the radicle was used, ensuring standardization of early growth evaluation. Samples were oven-dried at 65 °C for 48 h until constant mass, then weighed on an analytical balance with 0.0001 g precision. The mean dry weight per sample unit was obtained by dividing the total dry mass by the number of seedlings or radicles analyzed, as appropriate.

### 2.7. Growth Promotion Assay

For the growth promotion tests, only species whose seeds were inoculated with the bacteria showed superior germination performance to those not inoculated were selected. Seeds were sown in a non-sterilized cultivation substrate, placed in 300 mL plastic pots, and maintained at 26 °C under continuous light (24 h photoperiod) and relative humidity of 60–65%. Four days after sowing, the substrate was saturated with 10 mL of bacterial culture grown for 10 days in a medium containing 2% sucrose and 1% yeast extract under ambient conditions (~25 °C) [72]. Negative controls received either sterile distilled water or the same sucrose–yeast extract broth without bacterial inoculation. The substrate consisted of sphagnum peat, expanded vermiculite, dolomitic limestone, agricultural gypsum, NPK fertilizer, and micronutrients, with a pH of 5.0. The experiment lasted 30 days, during which the bacterial suspension was applied three times: the first on day 4 (post-sowing), the second at day 14, and the third two days before the end of the experiment (day 28). In each application, the controls were treated with either sterile water or the uninoculated broth. Each experimental unit consisted of two to three plants, with ten replicates per treatment. All plants were maintained in a B.O.D. chamber at 26 °C, 60–65% humidity, and continuous light throughout the experiment. The 24 h photoperiod was chosen to enhance vegetative growth [73]. To assess the growth-promoting capacity, we measured fresh and dry leaf weight, total leaf area (cm^2^), specific leaf area (SLA), chlorophyll content (a, b, and total carotenoids), and dry weight of both shoot and root tissues [74,75,76].

### 2.8. Assessment of Growth Promotion Capacity

For leaf dry mass determination, the second pair of fully expanded leaves was used, with one leaf collected from each experimental unit per treatment. The samples were properly labeled, and their fresh weight was first recorded using an analytical balance with a precision of 0.0001 g. The same leaves, identified by corresponding sample codes, were subsequently used to measure leaf area (cm^2^), dry mass, and specific leaf area (SLA). Leaf area was determined by scanning each leaf alongside a 1 cm^2^ reference scale, and the images were analyzed using the ImageJ software (v.1.8.0) [77]. Subsequently, the leaves were dried in an oven at 75 °C for 72 h, and the dry weight was measured on the same analytical balance. SLA was calculated as the ratio of measured leaf area by its corresponding dry weight, with results expressed in cm^2^ g^−1^ [74,76].

To determine photosynthetic pigment concentrations, four independent sample units were selected per treatment. From each sample unit, two leaf discs, each 0.55 cm in diameter, were collected and immediately placed into amber microtubes containing 2 mL of N, N-dimethylformamide (DMF) solvent. The samples were then incubated in the dark and refrigerated (4 °C for 48 h). The supernatant was then subjected to spectrophotometric readings at 663.8 and 646.8 nm for chlorophylls a and b, and at 480 nm for total carotenoids. Pigment concentrations (mg·mL^−1^) were calculated following the equations proposed by Lichtenthaler and Wellburn [78] and subsequently converted to mg of pigment per cm^2^ of leaf area to standardize the results according to the sampled area. To assess the shoot and root dry biomass, the organs were gently separated, rinsed with distilled water, and oven-dried at 75 °C for 48 h. Dry weight was determined on an analytical balance (precision 0.0001 g). As each replicate consisted of two to three plants, the average dry mass per plant was obtained by dividing the total value by the number of individuals.

### 2.9. In Vitro Antagonism Capacity Test

For the BACIII strain antagonism assay, we followed the dual culture method used by Ding et al. [79], Azeem et al. [80], and Lu et al. [81], with minor adaptations. The antagonistic activity was evaluated against 21 phytopathogens belonging to seven genera: *Alternaria* sp. (CCUB 3242), *Calonectria* sp. (CCUB 4874), *Calonectria* sp. (CCUB 4877), *Colletotrichum* sp. (CCUB 2209), *Colletotrichum* sp. (CCUB 3217), *Colletotrichum* sp. (CCUB 4985), *C. plurivorum* (CCUB 3183), *C. theobromicola* (CCUB 3680), *Corynespora cassiicola* (CCUB 2027), *C. cassiicola* (CCUB 2028), *C. cassiicola* (CCUB 2142), *C. cassiicola* (CCUB 2311), *Fusarium* sp. (CCUB 2853), *Fusarium* sp. (CCUB 2874), *Fusarium* sp. (CCUB 4634), *Fusarium* sp. (CCUB 4653), *Macrophomina euphorbiicola* (CCUB 4669), *M. phaseolina* (CCUB 3284), *M. pseudophaseolina* (CCUB 4681), *M. tecta* (CCUB 3329) and *Sclerotinia sclerotiorum* (CCUB 6127). The assays were conducted on a medium containing 2% sucrose and 1% yeast extract. Mycelial discs (0.5 cm^2^), obtained from Potato Dextrose Agar (PDA) cultures, were inoculated at one end of the plate and incubated at 24 ± 2 °C for 24 h. Subsequently, a stripe of the BACIII strain was applied to the opposite end. The control consisted of the phytopathogen inoculated into the culture medium alone. Each treatment was conducted with ten independent replicates. The experiment was monitored for up to 15 days, and was interrupted for each fungal isolate when mycelial growth reached less than 1 cm from the edge opposite the inoculation. The percentage inhibition (PI) of mycelial growth was calculated using the mean growth diameter of the control (C) and the treatment (T), as follows: PI (%) = [(C − T)/C] × 100. Images of fungal cultures were acquired and processed following the protocol described by dos Reis et al. [82].

### 2.10. Statistical Analysis

All statistical analyses were performed in R language (v. 4.4.0; R Core Team, Vienna, Austria, 2024) using RStudio (v. 2024.04.0) as the development environment, with the packages dplyr (v. 1.1.4), rstatix (v. 0.7.2), and car (v. 3.1-3). All figures were generated using ggplot2 (v. 3.5.2). Before the inferential analyses, data were examined for normality (Shapiro–Wilk test) and homogeneity of variances (Levene’s or Bartlett’s tests) to ensure the validity of subsequent procedures. For antagonism and germination assays, which involved comparisons between two groups, Student’s *t*-test was applied when the assumptions of normality and homogeneity were met (*p* > 0.05), while the Wilcoxon–Mann–Whitney test was applied when these assumptions were violated. Resulting *p*-values were adjusted for multiple testing using the Benjamini–Hochberg false discovery rate (FDR/BH) correction. For growth-promotion variables with multiple treatments, analyses were performed using Analysis of Variance (ANOVA), followed by Tukey’s HSD post hoc test when assumptions were met. When the assumption of normality was violated, the Kruskal–Wallis test was employed, followed by Dunn’s multiple comparison test with Bonferroni correction. Statistical significance was established at *p* ≤ 0.05.

## 3. Results

### 3.1. Sequencing Quality, Genome Assembly, and Annotation

**Figure 1:**
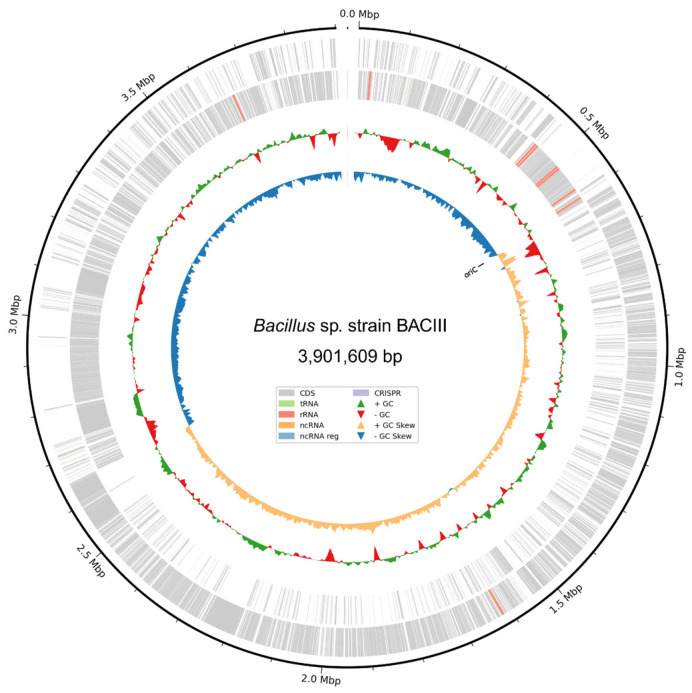
Circular representation of the *Bacillus siamensis* BACIII genome generated using the Bakta Web platform.

Sequencing yielded approximately 43,000 reads totaling 149 Mb (~33X genome coverage), with nearly all reads passing the quality filter (Supplementary Material S1—Supplementary Figure S1). The genome assembly of *Bacillus siamensis* BACIII resulted in a single complete chromosome with a total size of 3,901,609 bp (Figure 1), and no plasmids were identified. The GC content was 46.4%, with no ambiguous bases detected (N ratio = 0.0), and the coding density reached 89.9%. BUSCO analysis against the bacillales_odb10 dataset (450 orthologs) identified 449 complete BUSCOs (99.8%). A total of 4011 genomic features were identified. These included 3766 protein-coding sequences (CDSs), 37 pseudogenes, and 134 hypothetical proteins. In addition, 87 tRNAs, 27 rRNAs, one tmRNA, 29 ncRNAs, and 60 ncRNA regions were annotated. No CRISPR arrays, plasmid origins (oriV or oriT), or gaps were detected, while a single oriC region was identified. The oriC region was identified at approximately 0.6 Mbp. Four small open reading frames (sORFs) were also annotated. SignalP 6.0 identified a set of putatively secreted proteins predominantly classified as classical signal peptide (SP) proteins (*n* = 138), followed by lipoproteins (LIPO; *n* = 90). Alternative secretion pathways were rare, including Tat-dependent proteins (TAT; *n* = 3), pilin-like proteins (PILIN; *n* = 3), and a single Tat-associated lipoprotein (TATLIPO; *n* = 1).

### 3.2. Phylogenetic Identification and Analysis

TETRA analysis, which compares tetranucleotide frequency patterns to assess genome-wide compositional similarity, showed high and consistent z-scores (>99.75%) among closely related *Bacillus* species, with the highest value observed for *B. siamensis* KCTC 13613 (99.994), and slightly lower values for *B. velezensis* and *B. amyloliquefaciens* (Supplementary Material S1—Supplementary Table S1). The highest value was observed for *B. siamensis* KCTC 13613 (99.99%), while *B. velezensis* and *B. amyloliquefaciens* showed slightly lower but still high values. ANIb analysis revealed the highest nucleotide identity with *B. siamensis* (up to 97.73%), whereas *B. velezensis* and *B. amyloliquefaciens* presented lower values (~94% and ~93–94%, respectively), indicating clear separation among these taxa. Similarly, ANIm analysis confirmed this pattern, with the highest similarity observed for *B. siamensis* (98.11%), while the remaining species showed values around 94–95% (Supplementary Material S2).

**Figure 2:**
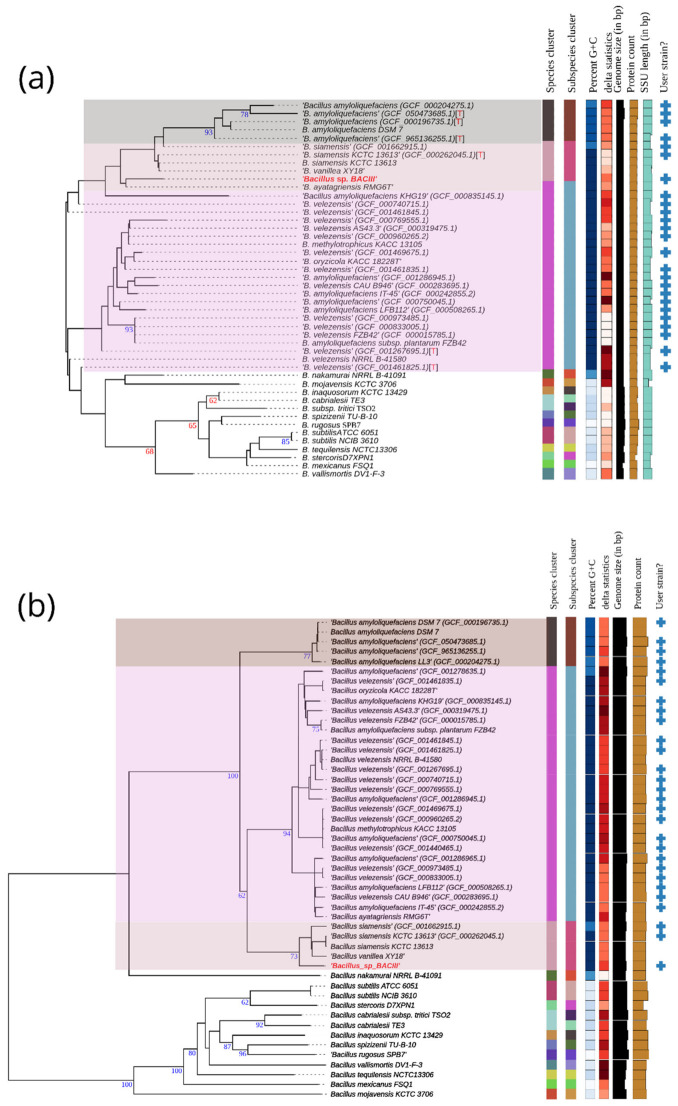
Phylogenetic analyses of *Bacillus siamensis* BACIII. (**a**) 16S rRNA gene-based phylogenetic tree showing the taxonomic placement of BACIII among closely related Bacillus species. (**b**) Genome-based phylogenetic tree generated using the Type (Strain) Genome Server (TYGS) based on whole-genome sequence data. Branch lengths are scaled according to the GBDP distance formula d5, and numbers above the branches indicate GBDP pseudo-bootstrap support values derived from 100 replicates. GenBank accession identifiers are shown in parentheses, with master record accessions truncated.

The analysis performed using the TYGS web server also indicates that strain BACIII belongs to *B. siamensis* (Figure 2). Digital DNA–DNA hybridization values between strain BACIII and the type strain *B. siamensis* KCTC 13613 ranged from 83.0% to 94.5%, depending on the dDDH formula applied (dDDH d0, d4, and d6) (Supplementary Material S2). The difference in G + C content between the genomes was lower than 0.6% (Supplementary Material S2). High dDDH values were also observed in comparisons with genomes assigned to *B. vanillea* (CGMCC 8629; NCCB 100507).

### 3.3. Clustering of Secondary Metabolites

**Figure 3:**
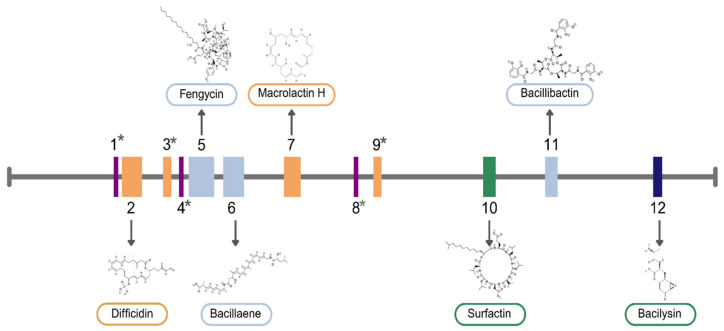
Biosynthetic gene clusters (BGCs) identified in the BACIII genome and their genomic orientation, as predicted by antiSMASH v8.0.4. The asterisk (*) indicates BGCs whose predicted products show no known similarity to characterized metabolites.

Genomic analysis identified 12 BGCs in the BACIII genome. These clusters include NRPS, PKS, T3PKS, hybrid NRPS/PKS systems, terpene and terpene-precursor regions, as well as BGCs classified as “other” or PKS-like. Seven clusters displayed high similarity to characterized biosynthetic pathways. These correspondences include difficidin (transAT-PKS), fengycin (NRPS), bacillaene (hybrid NRPS/PKS), macrolactin H (PKS), surfactin (NRPS), bacillibactin (NRPS), and bacilysin (other) (Figure 3; Supplementary Material S1—Supplementary Table S2). The remaining clusters comprise terpenoid or terpenoid-precursor regions, T3PKS clusters, and a PKS-like locus with no high-confidence match.

### 3.4. Genomic Island Analysis

Genomic island analysis identified five regions distributed along the chromosome, with sizes ranging from approximately 5.1 kb to 34.2 kb (Supplementary Material S1—Supplementary Figure S2; Supplementary Material S3). Most islands were predicted by the IslandPath-DIMOB method, with additional detection by SIGI-HMM. The largest genomic island (~34.2 kb) comprised mainly hypothetical proteins, along with annotated genes including immA, immR, and xis (mobility-related genes). Additional genes identified in this region included rapI, bcrA, norG, yjcF, and a carboxylate/amino acid/amine transporter (regulatory, resistance, and transport-related functions). A smaller island (~5.1 kb), predicted by SIGI-HMM, contained rlmH and a CxxH/CxxC protein, with the remaining genes annotated as hypothetical proteins. Another island (~7.3 kb) was composed predominantly of hypothetical proteins, along with padC, paiA, and an IS1595-family transposase (metabolism and mobility-related genes). A genomic island of approximately 15.9 kb contained genes such as rph, hsdM, hsdS, ilvB, and ilvH, along with additional genes annotated as hypothetical proteins and enzymes involved in nucleotide metabolism (including restriction-modification system components). The final island (~18.2 kb) contained genes including ybiV, ribZ, gdhIV, glcU, azoR2, yvgN, and CueR, as well as several hypothetical proteins (associated with metabolic and stress-related functions).

### 3.5. Analysis of Genomic Functional Characteristics

Functional annotation of the *Bacillus siamensis* BACIII genome using BlastKOALA assigned functions to 2275 genes, corresponding to 60.8% of the predicted coding sequences. The most abundant categories were protein families related to genetic information processing, signaling, and cellular processes, followed by pathways involved in carbohydrate, amino acid, nucleotide, lipid, and cofactor metabolism (Supplementary Material S1—Supplementary Figure S3). Genes associated with environmental information processing were also frequent, including components related to transport and regulatory systems. KEGG pathway mapping showed the presence of genes involved in central carbon metabolism, amino acid biosynthesis and degradation, energy metabolism, and cofactor and vitamin biosynthesis (Supplementary Material S1—Supplementary Figure S3).

### 3.6. Comparative Genomics

**Figure 4:**
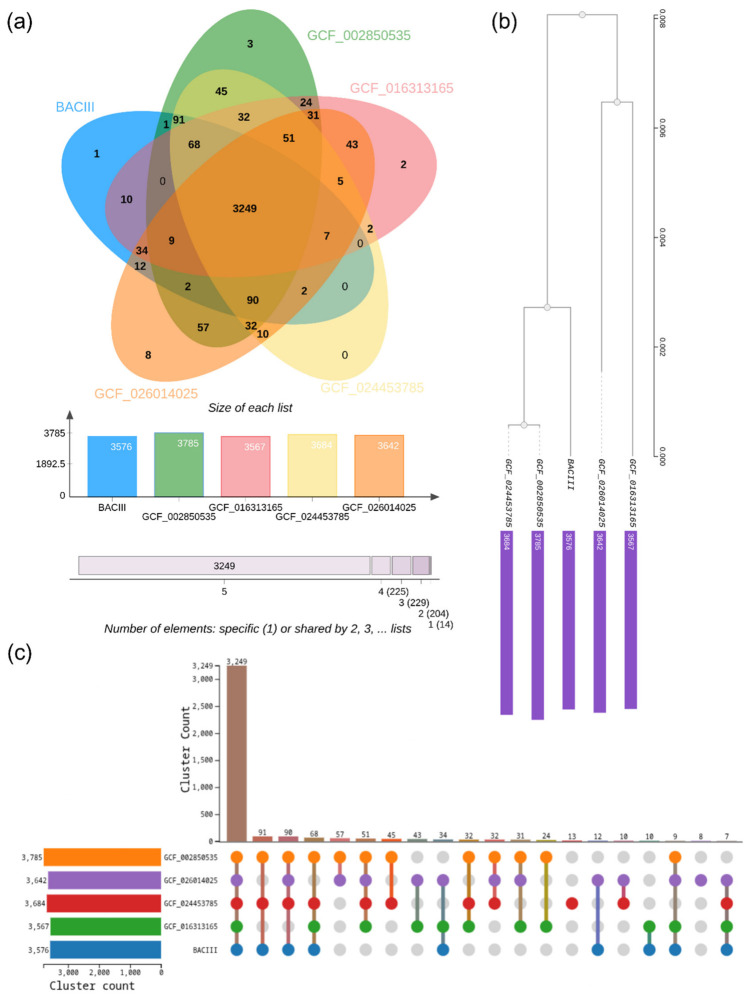
Pangenome structure and genomic conservation among *Bacillus siamensis* genomes and the *Bacillus siamensis* BACIII strain. (**a**) Venn diagram showing the distribution of gene clusters shared among the five genomes, highlighting the conserved core and genome-specific clusters. The bar plot below indicates the total number of gene clusters in each genome. (**b**) Hierarchical clustering based on shared gene content, illustrating the genomic similarity among the analyzed genomes. (**c**) UpSet plot summarizing the number of gene clusters unique to each genome and shared by different combinations of two, three, four, or all five genomes, with bars indicating cluster counts and dots representing genome intersections. GCF_051628075: *Bacillus siamensis* M54 (reference genome); GCF_016313165: *Bacillus siamensis* B28; GCF_002850535: *Bacillus siamensis* SCSIO 05746; GCF_024453785: *Bacillus siamensis* WYJ-E14.

Comparative analysis of the five genomes revealed a high degree of gene conservation, with a robust genomic core shared among all analyzed lineages. In total, 3249 gene clusters were shared among the five genomes, representing the largest fraction of the pangenome and indicating the maintenance of a highly conserved functional core (Figure 4). This core is mainly composed of genes associated with essential functions, including primary metabolism, basic cellular processes, and structural maintenance. Despite this conservation, the analysis also revealed variation in the accessory genome, with lineage-specific and partially shared sets of genes distributed among two, three, or four genomes. The number of exclusive clusters was relatively low across all lineages, ranging from 0 to 10 genes. Clusters shared by subsets of genomes, particularly those present in four genomes, totaled 225 clusters, whereas clusters shared by three and two genomes accounted for 229 and 204 clusters, respectively.

**Figure 5:**
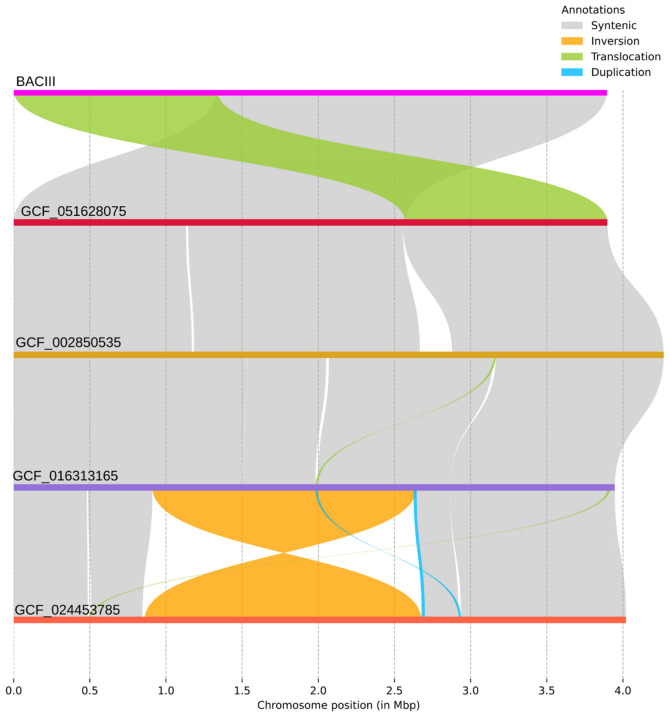
Synteny analysis among the five analyzed genomes, including *Bacillus siamensis* BACIII and related *Bacillus siamensis* strains. Whole-genome alignments were generated using the program syri, and the results were rendered using plotsr. The genome accessions are displayed at the left, with conserved regions (synteny) shown in gray, indicating high collinearity across genomes. Other types of structural variations are coloring according to the top-right legend. GCF_051628075: *Bacillus siamensis* M54 (reference genome); GCF_016313165: *Bacillus siamensis* B28; GCF_002850535: *Bacillus siamensis* SCSIO 05746; GCF_024453785: *Bacillus siamensis* WYJ-E14.

Synteny analysis further supported the high level of genomic conservation, revealing extensive collinearity among the analyzed genomes (Figure 5). Most regions were classified as syntenic, indicating strong conservation in gene order and orientation across the genomes. However, localized structural variations were also detected, including inversion and translocation events, particularly in the central regions of the chromosomes. A few duplication signals were observed, although at a lower frequency compared to other rearrangements.

Although genomic analyses have shown that the different strains share virtually the same core genes, BACIII and GCF_002850535 harbor 14 copies of this orthogroup (Cluster 1), whereas GCF_026014025 contains 10 copies, and the remaining strains have fewer than four copies. This cluster corresponds to a PKS family, enzymes involved in the synthesis of secondary metabolites. In addition, BACIII contained two copies of O34330 (ribonuclease YobL), whereas the other strains showed only a single copy.

### 3.7. Genes Related to Plant Growth Promotion

**Figure 6:**
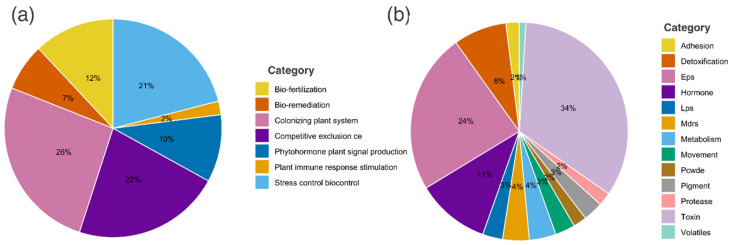
Functional classification of plant–microorganism interaction traits predicted for *Bacillus siamensis* BACIII using the PLabASE platform. (**a**) Distribution of plant growth-promoting traits based on PGPT-Pred. (**b**) Functional categorization based on PIFAR-Pred. Values are expressed as relative percentages.

Using the PLabASE platform, functions related to plant–microorganism interactions were predicted based on PGPT-Pred and PIFAR-Pred approaches (Figure 6). PGPT-Pred analysis revealed a predominance of functions associated with plant system colonization (26%), competitive exclusion (22%), and stress control/biocontrol (21%). Categories related to biofertilization (12%) and phytohormone/plant signal production (10%) were also well represented, whereas bioremediation (7%) and plant immune response stimulation (2%) were less frequent. In contrast, PIFAR-Pred analysis showed higher abundances of toxin-related genes (34%) and exopolysaccharide (EPS) biosynthesis (24%). Hormone-related functions (11%) and detoxification mechanisms (8%) were also identified. Other categories, including multidrug resistance (4%), metabolism (4%), LPS (3%), movement (3%), and pigments (3%), were present at lower frequencies, while adhesion, PCWDE, proteases, and volatile compounds occurred at low levels (≤2%).

### 3.8. Germination Tests

**Figure 7:**
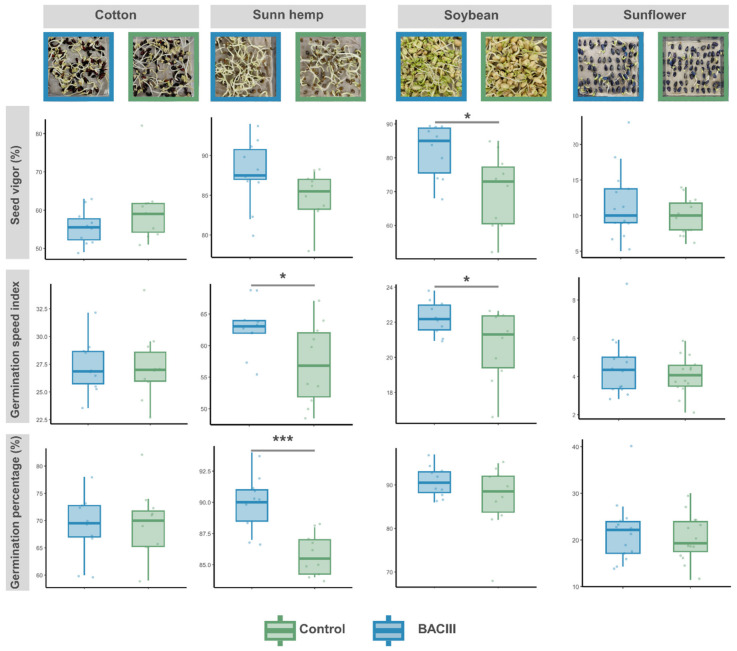
Effect of treatment with the *Bacillus siamensis* strain BACIII on seed germination of four agricultural plant species. The figure presents boxplots showing Seed Vigor (%), Germination Speed Index (GSI), and Germination Percentage (PG%) for seeds of cotton (*Gossypium hirsutum*, cv. FM 985 GLTP), sunn hemp (*Crotalaria juncea*, cv. C52333-C), soybean (*Glycine max*, cv. Brasmax Olimpo IPRO 80I82RSF), and sunflower (*Helianthus annuus*, cv. Hélio 251). Asterisks denote statistically significant differences between treatments within the same species and parameter (*p* ≤ 0.05), based on Student’s *t*-test or Wilcoxon–Mann–Whitney test: “*” *p* = 0.05–0.02; “***” *p* = 0.001. Sample size per treatment: *n* = 10 for cotton; *n* = 10 for sunn hemp; *n* = 5 for soybean; *n* = 14 for sunflower.

We evaluated four plant species treated with and without the BACIII strain. Overall, statistically significant differences (*p* ≤ 0.05) were observed in soybean and sunn hemp (Figure 7). For cotton and sunflower, we found no significant differences (*p* > 0.05) across the three metrics evaluated. Cotton showed no change, with PG values of 69.4 ± 6.24% in the control and 68.9 ± 5.70% with BACIII. The IVG was similarly unaffected (control 27.3 ± 3.15 vs. BACIII 27.3 ± 2.42). Likewise, sunflower did not respond to the treatment, with a PG of 20.4 ± 5.12% in the control group versus 21.6 ± 6.67% in the treatment group. In contrast, BACIII induced significant improvements in sunn hemp, with PG increasing from 85.8 ± 1.62% (control) to 90.0 ± 2.21% (BACIII), and IVG rising from 57.1 ± 6.48% to 62.8 ± 4.21%. For soybean, significant improvements were observed in VS, which increased from 70.1 ± 11.01% (control) to 82.1 ± 7.68% (BACIII), and in IVG, which rose from 20.7 ± 2.02 to 22.2 ± 0.96. The PG for soybean did not show a statistically significant difference (86.9 ± 7.99% in the control vs. 90.8 ± 3.52% in BACIII). At the end of the experiment, we measured the dry weight of sunn hemp and cotton seedlings and of soybean and sunflower radicles. Overall, no significant differences were observed among treatments for most species, except for soybean, which showed a significantly higher radicle dry weight in the BACIII treatment (Supplementary Material S1—Supplementary Figure S4).

### 3.9. Plant Growth-Promoting Capacity

For growth promotion tests, we used only the species that showed positive results in bacterial inoculation during the germination test: sunn hemp and soybean. We evaluated several leaf traits, including leaf area (cm^2^), SLA, fresh and dry weight, and water content (Supplementary Material S1—Supplementary Figure S5). For sunn hemp, treatment with the BACIII strain induced significant differences (*p* < 0.001) in leaf area and in both fresh and dry weight. Water percentage and SLA did not differ significantly between treatments. In leaf area, the BACIII treatment was significantly higher than control I and control II (*p* < 0.001), with a mean value of 8.50 ± 1.70 cm^2^. In this case, control II (5.60 ± 1.27 cm^2^), which consisted only of the culture medium without bacteria, was also superior to control I (3.73 ± 0.95 cm^2^), which was inoculated only with water. For leaf fresh weight, the BACIII treatment (0.154 ± 0.039 g) was likewise significantly greater (*p* < 0.0001) than controls I (0.065 ± 0.017 g) and II (0.096 ± 0.026 g). For dry leaf weight, we also observed significant differences (*p* < 0.0001) between BACIII (0.0144 ± 0.0033 g) and the controls, with control II (0.0102 ± 0.0031 g) being significantly greater than control I (0.0069 ± 0.0020 g). Water percentage was similar among treatments (BACIII: 90.55 ± 1.20%; Control I: 89.28 ± 1.45%; Control II: 89.42 ± 1.41%). Finally, for soybean, no significant differences were observed in any of the leaf traits evaluated.

**Figure 8:**
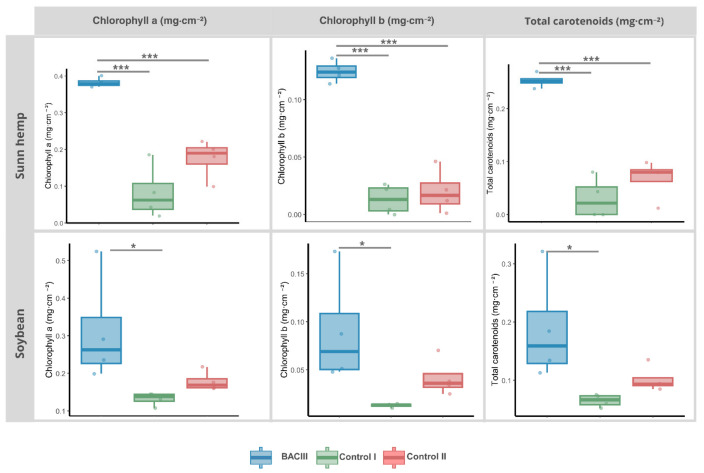
Effect of *Bacillus siamensis* strain BACIII treatment on leaf pigment contents of two agricultural plant species. Chlorophyll a, chlorophyll b, and total carotenoids (mg·cm^−2^) in sunn hemp (*Crotalaria juncea*, cv. C52333-C) and soybean (*Glycine max*, cv. Brasmax Olimpo IPRO 80I82RSF) under three treatments: BACIII (bacterial inoculation), Control I (water only), and Control II (culture medium without bacteria). Asterisks denote statistically significant differences between treatments within the same species and parameter (*p* ≤ 0.05), based on ANOVA followed by Tukey’s HSD test or Kruskal–Wallis followed by Dunn’s multiple comparison test with Bonferroni correction:“*” *p* = 0.05–0.02;; “***” *p* = 0.001. Sample size per treatment: *n* = 4 for sunn hemp; *n* = 4 for soybean.

Regarding leaf pigment content, the two species exhibited distinct responses to treatment with the BACIII strain. In sunn hemp, BACIII treatment induced a highly significant increase (*p* < 0.0001) in all pigments compared with both controls (Figure 8). Leaf chlorophyll a reached 0.382 ± 0.013 under BACIII, which was higher than in Control I (0.083 ± 0.073) and Control II (0.175 ± 0.053). A similar pattern was observed for chlorophyll b (BACIII: 0.124 ± 0.009; Control I: 0.013 ± 0.013; Control II: 0.020 ± 0.019) and total carotenoids (BACIII: 0.253 ± 0.013; Control I: 0.031 ± 0.039; Control II: 0.067 ± 0.038). In soybean, BACIII also increased leaf pigment levels, with values significantly higher than those of Control I (water only) (*p* < 0.05). However, no statistical difference was detected when compared with Control II (culture medium).

**Figure 9:**
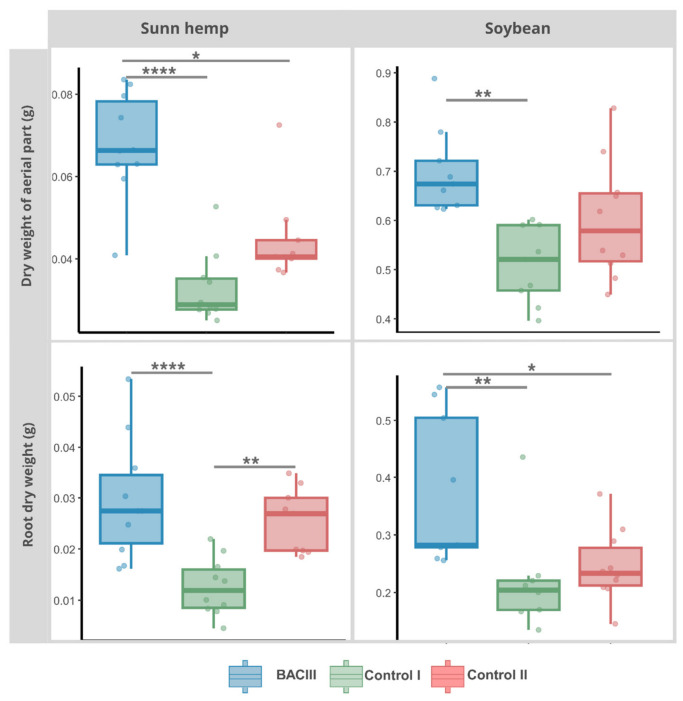
Effect of *Bacillus siamensis* BACIII treatment on the dry weight of shoots and roots of two agricultural plant species. The figure presents shoot dry weight (g) and root dry weight (g) of sunn hemp (*Crotalaria juncea*, cv. C52333-C) and soybean (*Glycine max*, cv. Brasmax Olimpo IPRO 80I82RSF) under three treatments: BACIII (bacterial inoculation), Control I (water only), and Control II (culture medium without bacteria). Asterisks denote statistically significant differences between treatments within the same species and parameter (*p* ≤ 0.05), based on ANOVA followed by Tukey’s HSD test or Kruskal–Wallis followed by Dunn’s multiple comparison test with Bonferroni correction: “*” *p* = 0.05–0.02; “**” *p* = 0.01; “****” *p* = 0.0001. Sample size per treatment: *n* = 9 for soybean in BACIII, Control_I, and sunn hemp Control_II; *n* = 10 for sunn hemp in BACIII and Control_I, and soybean Control_I.

Differences in shoot and root dry weight were evident between treatments, with contrasting responses in the two species (Figure 9). In sunn hemp, shoot dry weight under BACIII treatment (0.0679 ± 0.0129 g) was significantly higher than in Control I (*p* < 0.0001; 0.0381 ± 0.0051 g) and Control II (*p* < 0.05; 0.0519 ± 0.0084 g). For root dry weight, significant differences were found between BACIII (0.0296 ± 0.0119 g) and Control II (0.0195 ± 0.0044 g) when compared with Control I (0.0093 ± 0.0039 g), while BACIII and Control II did not differ statistically. In soybean, BACIII also promoted higher shoot dry weight (0.699 ± 0.087 g) than Control I (0.509 ± 0.077 g), with no difference from Control II (0.600 ± 0.120 g). Root dry weight was significantly higher (*p* < 0.05) under BACIII (0.373 ± 0.129 g) compared with both controls (Control I: 0.219 ± 0.087 g; Control II: 0.246 ± 0.063 g).

### 3.10. In Vitro Inhibition of Phytopathogens

**Figure 10:**
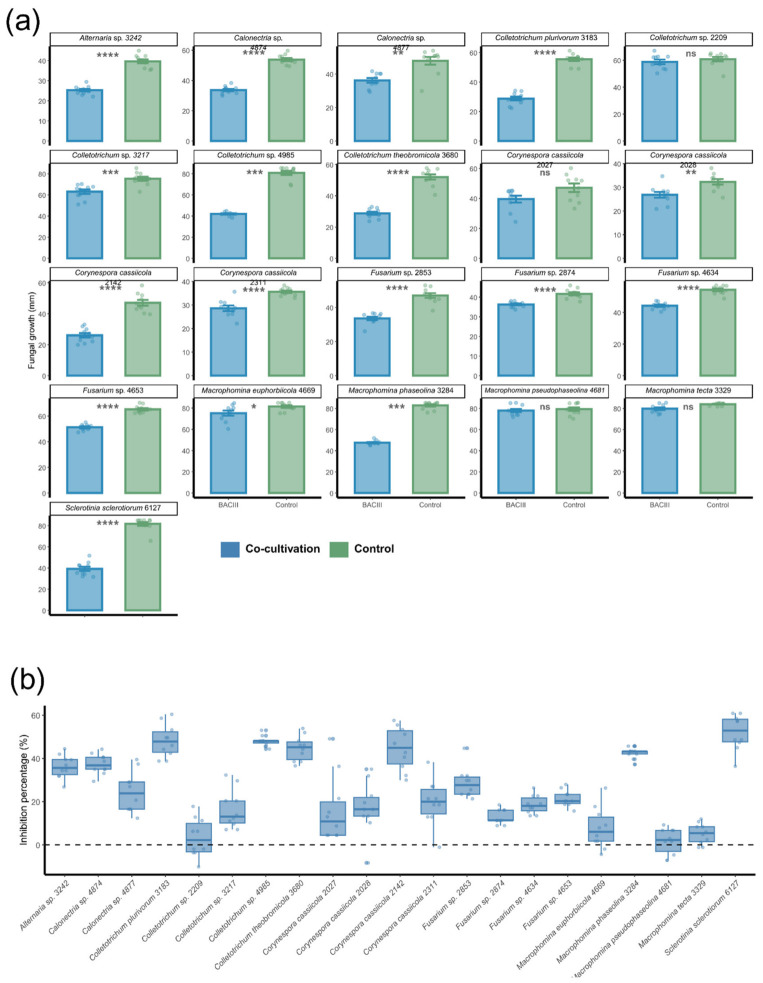
Results of the coculture inhibition assays between *Bacillus siamensis* strain BACIII and different phytopathogenic fungi. (**a**) Comparison between the radial growth of each fungal pathogen in monoculture and in coculture with strain BACIII. (**b**) Percentage of growth inhibition calculated for each fungal species when exposed to BACIII. Asterisks denote statistically significant differences between treatments within the same species and parameter (*p* ≤ 0.05), based on ANOVA followed by Tukey’s HSD test or Kruskal–Wallis followed by Dunn’s multiple comparison test with Bonferroni correction: “*” *p* = 0.05–0.02; “**” *p* = 0.01; “***” *p* = 0.001; “****” *p* = 0.0001. Sample size per treatment: *n* = 10.

The dual culture assay demonstrated that the *Bacillus siamensis* BACIII strain possesses a broad spectrum of antifungal activity, inhibiting the mycelial growth of 17 out of 21 tested phytopathogens (approximately 81%), which belong to seven distinct genera (Figure 10; Supplementary Material S1—Supplementary Figure S6 and Table S3). The percent inhibition (PI%) varied among the phytopathogens. The most prominent efficacy was observed against the pathogen *Sclerotinia sclerotiorum* CUUB 6127, with a mean inhibition of 52.02 ± 8.09%. High inhibition was also observed against the genus *Colletotrichum*, with the BACIII strain inhibiting *C. plurivorum* CUUB 3183 (48.11 ± 7.59%) and *Colletotrichum* sp. CUUB 4985 (48.02 ± 2.41%). However, activity was inconsistent within the genus, as BACIII was ineffective against *Colletotrichum* sp. CUUB 2209, which recorded a PI% of only 3.29 ± 8.90%. This variability was also evident in tests against *Macrophomina*: the isolate *M. phaseolina* CUUB 3284 was inhibited (42.51 ± 2.56%), while *M. pseudophaseolina* CUUB 4681 (1.64 ± 6.06%) and *M. euphorbiicola* CUUB 4669 (7.62 ± 9.49%) showed little or no sensitivity. The phytopathogens of the genera *Alternaria* (*Alternaria* sp. CUUB 3242, 36.11 ± 5.33%) and *Calonectria* (*Calonectria* sp. CUUB 4874, 37.36 ± 4.64%) showed moderate growth inhibition. The genera *Corynespora* and *Fusarium* exhibited the greatest heterogeneity of response. Inhibition in *Corynespora* varied widely, from moderate activity in *C. cassiicola* CUUB 2027 (16.14 ± 15.38%) to promising results in *C. cassiicola* CUUB 2142 (44.59 ± 9.83%). Similarly, inhibition in *Fusarium* ranged from 12.95 ± 3.40% (*Fusarium* sp. CUUB 2874) to 28.51% ± 6.89% (*Fusarium* sp. CUUB 2853).

## 4. Discussion

In this study, we investigated a *Bacillus siamensis* BACIII strain using an integrated approach that combined genomic analyses with phenotypic assays of plant growth promotion and antagonism against phytopathogenic fungi. The results provide a comprehensive view of the genomic organization of this strain and its functional characteristics, enabling inferences about mechanisms underlying plant interactions and the biological control of fungal pathogens. This integration of genomic data and experimental evidence has advanced the understanding of interactions between plant growth-promoting bacteria and their plant hosts, as well as the identification of genetic traits related to antimicrobial activity [35,83,84]. Such combined approaches have been increasingly employed in the characterization of *Bacillus* strains, as they allow a more consistent interpretation of the relationship between genetic content and observed functional performance [85,86,87].

### 4.1. Quality of Sequencing and Taxonomic Classification

The results of genome quality analyses demonstrate the robustness of the sequencing and assembly obtained for *Bacillus siamensis* BACIII. Completeness estimates based on conserved marker genes, evaluated using both CheckM and BUSCO, indicate that the recovered genome comprehensively represents the expected gene content for strains of the genus [88,89]. The high completeness observed, together with the absence or only residual levels of contamination and redundancy, suggests that the assembly corresponds to a single, well-resolved genome. Given the high quality of the genome, multiple approaches were employed for the taxonomic delimitation of the BACIII strain. Analysis based on the 16S rRNA gene sequence revealed high similarity to *B. velezensis*. However, this species belongs to the so-called *B. amyloliquefaciens* operational group within the *B. subtilis* species complex, which includes closely related taxa such *as B. amyloliquefaciens*, *B. siamensis*, and *B. velezensis* [29,90]. The use of the 16S rRNA gene alone is insufficient for reliable species-level delimitation [91]. Phylogenomic analyses based on ANI, TETRA, and dDDH consistently placed the BACIII strain within the *B. siamensis* lineage. The ANI-based approach, in particular, is widely recognized as a robust method for species delimitation using whole-genome data [92,93,94]. In agreement with these results, the phylogenomic tree generated by TYGS grouped the BACIII strain within the *B. siamensis* clade, clustering on the same branch as *B. vanillea*, a heterotypic synonym of *B. siamensis* [95], further supporting the taxonomic assignment.

The high genomic similarity between the BACIII strain and *B. siamensis* lineages was also corroborated by pangenome analyses. Most genes were grouped into homologous families, whereas a smaller fraction corresponded to exclusive genes, characterizing a pangenome composed of a well-conserved genetic core and a relatively restricted accessory set. This degree of genomic sharing is consistent with previous observations for species belonging to the *B. amyloliquefaciens* Operational Group, including *B. velezensis* and *B. siamensis*, which exhibit high gene conservation associated with central metabolic and ecological functions [84,96,97,98]. Despite this high level of homology, genes classified as singletons and genes not belonging to the genomic core were also identified in the BACIII genome. These unique or low-frequency genes may confer strain-specific traits and are potentially associated with adaptive processes, ecological specialization, or responses to particular selective pressures, reflecting evolutionary mechanisms acting at the lineage level [99,100]. Pangenome-scale studies have shown that *Bacillus* species within the subtilis complex possess open pangenomes, suggesting ongoing gene acquisition and diversification linked to adaptation to different environmental niches [83,96,101]. However, it is important to note that the number of available genomes for *B. siamensis* remains considerably smaller than that for *B. velezensis* [96]. Consequently, the proportion of genes classified as singletons may be overestimated due to the limited dataset. As additional genomes are incorporated into future analyses, some of these genes are expected to be reassigned to the shared accessory genome, allowing a more accurate delineation of core, accessory, and lineage-specific gene fractions.

### 4.2. Functional Genomic Characteristics, Growth Promotion, and Inhibition of Phytopathogens

Functional annotation and metabolic reconstruction analyses indicate that the genome of *Bacillus siamensis* BACIII encodes a broad and well-integrated metabolic repertoire, including genes associated with genetic information processing, signaling, and cellular processes, reflecting a metabolically active genome equipped with complex regulatory systems. The presence of transporters, phosphotransferase systems, and two-component regulatory systems reinforces the strain’s ability to perceive and respond efficiently to environmental fluctuations. This metabolic versatility observed at the genomic level has already been reported for other *B. siamensis* strains [87,102,103] and can be largely explained by the association of *B. siamensis* with environments such as soil [104], the rhizosphere [105], or plant tissues as an endophyte [106]. These ecological niches are highly heterogeneous, subject to constant environmental changes, and characterized by intense competition for resources among microorganisms [107,108,109,110,111]. The BACIII strain was isolated as a root endophyte from soybean plants without disease symptoms in an area with a high incidence of charcoal rot. This environment can be considered heterogeneous and competitive, requiring versatile adaptive and metabolic capabilities, as observed in the genome of *Bacillus siamensis* BACIII.

Another relevant genomic feature associated with metabolic versatility and adaptive potential is the presence of genomic islands [109,110,111,112,113,114,115]. In the BACIII genome, genomic islands associated with mobile genetic elements such as transposases and recombinases were identified, suggesting dynamic genomic regions that may influence important adaptive traits [111,113]. Furthermore, the presence of restriction–modification systems, transcriptional regulators, and genes related to stress response and transport within these regions points to a role of these islands in adaptation to competitive environments and in conferring specific advantages in nutrient acquisition or in interactions with other organisms [111,113,114].

The BACIII bacterial strain was also able to reduce germination time and promote plant growth in sunn hemp and soybean. These results partially support the initial hypothesis of this study, as neither germination nor growth was promoted in cotton or sunflower. Plant growth promotion is a well-documented trait among *Bacillus* species belonging to the *B. amyloliquefaciens* Operational Group within the *B. subtilis* complex [35,87,102,104,105,106,115]. More specifically, different *B. siamensis* strains have been shown to promote the growth of plant species from distinct botanical families [87,102,104,105,106,116,117]. However, most of these studies evaluate growth promotion in only a single plant species, often from a single botanical family, which limits broader conclusions regarding host dependency. In this context, the results obtained here, based on germination, biomass, and chlorophyll content under the tested experimental conditions, suggest that the growth-promoting capacity of *Bacillus siamensis* BACIII is dependent on the plant species. It is noteworthy that the plants showing positive responses to inoculation in our study belong to the family Fabaceae, a botanical group widely recognized for establishing close and functional interactions with soil microorganisms, including rhizobacteria and nitrogen-fixing bacteria [118,119]. This may be related to differences in root exudate composition, root architecture, and the ability to recruit and sustain beneficial microorganisms, factors that can influence bacterial colonization and the expression of plant growth-promoting traits.

The observed variation in the capacity for interaction and plant growth promotion among plant species may be related to multiple factors, including, for example, the production of effector proteins [120,121]. PLABase results suggest that colonization and competitive capabilities may contribute to the establishment of interactions with the host plant. Based on genomic analyses, the BACIII strain exhibited a substantial repertoire of proteins with classical signal peptides, indicating a considerable investment in extracellular secretion mechanisms. These secreted effectors play a central role in mediating interactions between microorganisms and plants, being associated both with direct plant–bacterium interaction processes [9,122,123] and induction of physiological responses in plants, such as increased stress tolerance and improved vegetative development [13,124]; see the review by Xiao et al. [125]. The functional specificity of many of these proteins may help explain the restricted interaction spectrum and the plant species dependence observed in this study. Another relevant genomic characteristic is the presence of genomic islands, which may harbor genes associated with ecological adaptation and may include determinants involved in plant growth promotion [113]. Furthermore, it should be considered that inoculation with *B. siamensis* strains can lead to changes in the taxonomic profile of soil and rhizosphere microbial communities [105]. In this study, the bacterium was inoculated into plants grown in non-sterilized soil, which may have indirectly favored beneficial microorganisms that contributed to plant growth stimulation. Together, these genomic characteristics suggest that the outcome of the interaction depends not only on the bacterium itself, but also on the compatibility established with each host plant and its associated microbiota. Nevertheless, the presence of biosynthetic gene clusters does not necessarily imply active metabolite production under all environmental conditions, and further transcriptomic, metabolomic, or targeted chemical analyses will be required to confirm the expression and ecological relevance of these pathways.

In addition to its plant growth-promoting capabilities, the BACIII strain showed significant antagonistic activity against various phytopathogenic fungi. This result is consistent with one of the study’s hypotheses, as a broad spectrum of antimicrobial activity is a well-documented characteristic of *B. siamensis* strains [116,117,126]. From a practical standpoint, the results obtained are particularly relevant, as the strain was able to reduce the growth of different species belonging to the genera *Colletotrichum*, *Fusarium*, *Corynespora*, *Macrophomina*, *Alternaria*, and *Calonectria*, as well as the soilborne pathogen *S. sclerotiorum*. These phytopathogens have a high potential to cause severe losses in many agricultural crops and are widely recognized as agents of economically important diseases [127,128,129,130,131]. Controlling these pathogens with microorganisms is considered strategic for the sustainability of agricultural systems [132,133]. The inhibitory capacity observed for *Bacillus siamensis* BACIII is even more relevant given the increasing search for microorganisms that can reduce or replace the use of synthetic pesticides, an approach widely discussed in recent reviews on biological control [134,135]. The use of microorganisms as biological control agents has proven to be a promising strategy, including different *B. siamensis* strains already described in the literature [116,117,126]. On the other hand, despite the broad spectrum of inhibition observed, some fungi were not affected by the BACIII strain, indicating that the antagonistic activity may be species-dependent or even strain-dependent, reinforcing the complexity of the microbial interactions involved. In addition, the dual-culture assays employed in this study evaluate the overall antagonistic phenotype but do not distinguish between inhibition mediated by diffusible metabolites, volatile compounds, nutrient competition, or direct microbial interactions.

The broad spectrum of inhibitory activity observed may be associated with distinct antagonistic mechanisms, including competition for nutrients and ecological niches, as well as the production of secondary metabolites with antifungal activity [133,136]. Functional genome annotation revealed a complex genetic repertoire that likely underpins the metabolic versatility of the BACIII strain and its ability to compete effectively with other microorganisms in shared environments. The detection of twelve BGCs provides strong genomic support for the antagonistic potential observed in vitro. Rather than relying on a single antifungal compound, this repertoire suggests that BACIII possesses the capacity to produce multiple metabolites with complementary modes of action, potentially broadening its inhibitory spectrum. Among these clusters, seven showed similarity to those involved in the biosynthesis of difficidin, bacillaene, fengycin, surfactin, macrolactin H, bacillibactin, and bacilysin. Several of these compounds have been previously associated with antifungal activity. Difficidin and bacillaene have been reported to inhibit the growth of *Fusarium graminearum* [137]. The lipopeptides fengycin and surfactin exhibit activity against fungi from distinct taxonomic groups [138,139], including species of *Monilinia* and *Aspergillus* [140]. Macrolactins have been described as effective in reducing the growth of *Botrytis cinerea* [141], *Fusarium oxysporum*, and *Rhizoctonia solani* [142]. In contrast, bacillibactin has been primarily associated with antibacterial activity, reflecting its role in iron acquisition and competitive interactions within microbial communities [143,144]. Furthermore, with the expansion of the PKS family, enzymes involved in secondary metabolite synthesis may confer greater biosynthetic capacity on the strain [145,146]. Such heterogeneity in antagonistic mechanisms may contribute to the variation observed in the inhibition profiles, as individual fungal lineages may differ in their sensitivity to specific compounds or combinations of metabolites. Taken together, this biosynthetic repertoire suggests that the BACIII strain employs multiple inhibitory strategies mediated by the production of bioactive secondary metabolites. Such heterogeneity in antagonistic mechanisms may contribute to variation in inhibition profiles, as individual fungal lineages may differ in their sensitivity to specific compounds or combinations of metabolites.

## 5. Conclusions

Overall, the results presented here indicate that *Bacillus siamensis* BACIII is a genetically and functionally versatile strain, whose ecological performance may be related to a combination of genomic characteristics and strain-specific traits. The integration of high-quality genomic data with phenotypic assays enables consistent interpretation of its ability to interact with plants, promote growth in a host-dependent manner, and inhibit various phytopathogenic fungi. Together, these findings reinforce the relevance of *B. siamensis* BACIII as a promising candidate for sustainable agricultural applications and provide a solid foundation for future studies to elucidate the regulation, expression, and ecological roles of its bioactive compounds under field conditions.

## Data Availability

Genome Shotgun project has been deposited in DDBJ/ENA/GenBank under accession number JBTIYL000000000. The genome assembly described in this study corresponds to version JBTIYL010000000.

## Supplementary Materials

The following supporting information can be downloaded at: https://www.mdpi.com/article/10.3390/microorganisms14071569/s1. Supplementary Material S1 include Figures S1–S6 and Tables S1–S3. Figure S1. Density contour plot showing the distribution of basecalled read lengths and PHRED quality scores. Figure S2. Circular map generated by IslandViewer4 representing the locations of genomic islands within the genome of *Bacillus* sp. strain BACIII. Figure S3. Prediction of functional characteristics encoded in the *Bacillus* sp. BACIII genome. In (a–c) functional annotation obtained with BlastKOALA. Figure S4. Effect of *Bacillus* sp. strain BACIII treatment on seedling dry weight of four agricultural plant species. Figure S5. Effect of *Bacillus* sp. strain BACIII treatment on leaf morphological traits of two plant species. Figure S6. Double culture assay of the *Bacillus* sp. BACIII strain against phytopathogenic fungi. Table S1. Tetranucleotide (TETRA) frequency analysis of the *Bacillus* sp. strain BACIII genome. Table S2. Biosynthetic gene clusters identified in the *Bacillus* sp. strain BACIII genome and their closest known matches according to AntiSMASH (v8.0.4). Table S3. Results of statistical tests for the co-culture assay of the bacterial strain *Bacillus* sp. BACIII against different phytopathogenic fungi. Supplementary Material S2. Genome-based taxonomic identification of Bacillus siamensis strain BACIII. The workbook contains three worksheets summarizing the genomic similarity analyses used for taxonomic assignment: ANIb (Average Nucleotide Identity based on BLAST), ANIm (Average Nucleotide Identity based on MUMmer), and Type Strain Genome Server (TYGS) results, including the phylogenomic and digital DNA–DNA hybridization (dDDH) analyses performed against reference type strains. Supplementary Material S3. Predicted genomic islands identified in the genome of Bacillus siamensis strain BACIII. The workbook contains the genomic coordinates, prediction method, and annotation of genes located within each predicted genomic island.
