# Supplementary material for: Genomic and Functional Characterization of the Endophytic Bacillus siamensis Strain BACIII with Plant Growth-Promoting and Antifungal Activity
Source: Microorganisms. 2026 Jul 17;14(7):1569. doi: 10.3390/microorganisms14071569 (PMC13413643; doi:10.3390/microorganisms14071569)
Supplement: Supplementary file 1 [file microorganisms-14-01569-s001.zip › Supplementary material S1.pdf]

Genomic and functional characterization of the endophytic *Bacillus* strain BACIII with plant growth–promoting and antifungal activity

Jefferson Brendon Almeida dos Reis [1], Sofia Coradini Schirmer [1], Maria Regina Silveira Sartori da Silva [1], Andrei Stecca Steindorff [2], Patrícia Cardoso Cortelo [1], Georgios Joannis Pappas Jr [1] and Helson Mario Martins do Vale [1,*]

[1] University of Brasilia, Institute of Biological Sciences, Brasilia 70910-900, DF, Brazil
[2] US DOE Joint Genome Institute, Lawrence Berkeley National Laboratory, Berkeley, CA 94720, USA

* Correspondence: helson@unb.br

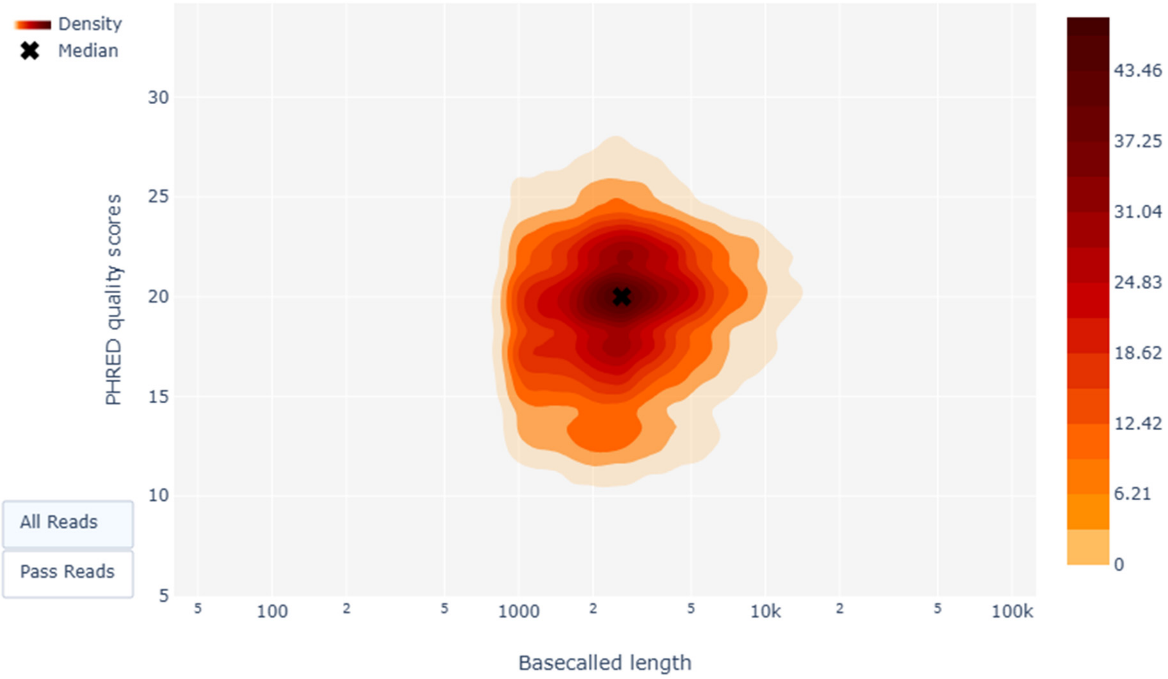

Supplementary Figure S1. Density contour plot showing the distribution of basecalled read lengths and PHRED quality scores. Warmer colors indicate regions of higher read density. The black cross marks the median values for both metrics.

Supplementary Table S1. Tetranucleotide (TETRA) frequency analysis of the *Bacillus* sp. strain BACIII genome

Genome	Strain	NCBI Ref Seq assembly	z-score
Bacillus velezensis [T]	NRRL B-41580	GCF_001461825.1	99.904
B. velezensis [T]	KCTC 13012	GCF_001267695.1	99.903
B. velezensis	KACC 18228	GCF_001461835.1	99.905
B. velezensis	NRRL B-4257	GCF_001461845.1	99.914
B. velezensis	NBIF-003	GCF_001440465.1	99.863
B. velezensis	FKM10	GCF_001469675.1	99.911
B. velezensis	JS25R	GCF_000769555.1	99.915

B. velezensis	AS43.3	GCF_000319475.1	99.913
B. velezensis	CAU B946	GCF_000283695.1	99.888
B. velezensis	FZB42	GCF_000015785.1	99.893
B. velezensis	L-H15	GCF_000833005.1	99.889
B. velezensis	L-S60	GCF_000973485.1	99.887
B. velezensis	KACC 13105	GCF_000960265.2	99.888
B. velezensis	NKYL29	GCF_000740715.1	99.889
B. siamensis	KCTC 13613	GCF_000262045.1	99.994
B. siamensis	SRCM100169	GCF_001662915.1	99.892
B. amyloliquefaciens **[T]**	DSM 7 = ATCC 23350 DSM 7	GCF_000196735.1	99.978
B. amyloliquefaciens **[T]**	CIP103265T	GCF_965136255.1	99.806
B. amyloliquefaciens **[T]**	NRRL B-14393	GCF_050473685.1	99.754
B. amyloliquefaciens	X1	GCF_000750045.1	99.91
B. amyloliquefaciens	Bs006	GCF_001278635.1	99.832
B. amyloliquefaciens	JRS5	GCF_001286945.1	99.891
B. amyloliquefaciens	JRS8	GCF_001286965.1	99.853
B. amyloliquefaciens	LL3	GCF_000204275.1	99.783
B. amyloliquefaciens	LFB112	GCF_000508265.1	99.882
B. amyloliquefaciens	KHG19	GCF_000835145.1	99.90
B. amyloliquefaciens	IT-45	GCF_000242855.2	99.870

[T] Strain type

Supplementary Table S2. Biosynthetic gene clusters identified in the *Bacillus* sp. strain BACIII genome and their closest known matches according to AntiSMASH (v8.0.4)

Region	Type	From	To	Similarity Confidence	Most similar known cluster	
Region 1	Terpene-precursor	588,083	608,973			
Region 2	TransAT-PKS	630,794	736,953	High	Difficidin	PKS
Region 3	T3PKS	854,485	895,585			
Region 4	Terpene	944,376	966,259			
Region 5	NRPS,betalactone,transAT-PKS	991,654	1,129,495	High	Fengycin	NRPS:Type I

Region 6	TransAT-PKS,NRPS,T3PKS	1,181,496	1,291,496	High	Bacillaene	NRPS:Type I+PKS:Type I
Region 7	TransAT-PKS	1,518,867	1,607,085	High	Macrolactin H	PKS
Region 8	Terpene	1,902,730	1,923,473			
Region 9	PKS-like	2,005,120	2,046,364			
Region 10	NRPS	2,600,593	2,666,000	High	Surfactin	NRPS:Type I
Region 11	Terpene-precursor,NRP-metallophore,NRPS,RiPP-like	2,943,983	3,009,321	High	Bacillibactin	NRPS:Type I
Region 12	Other	3,536,852	3,578,270	High	Bacilysin	other:other

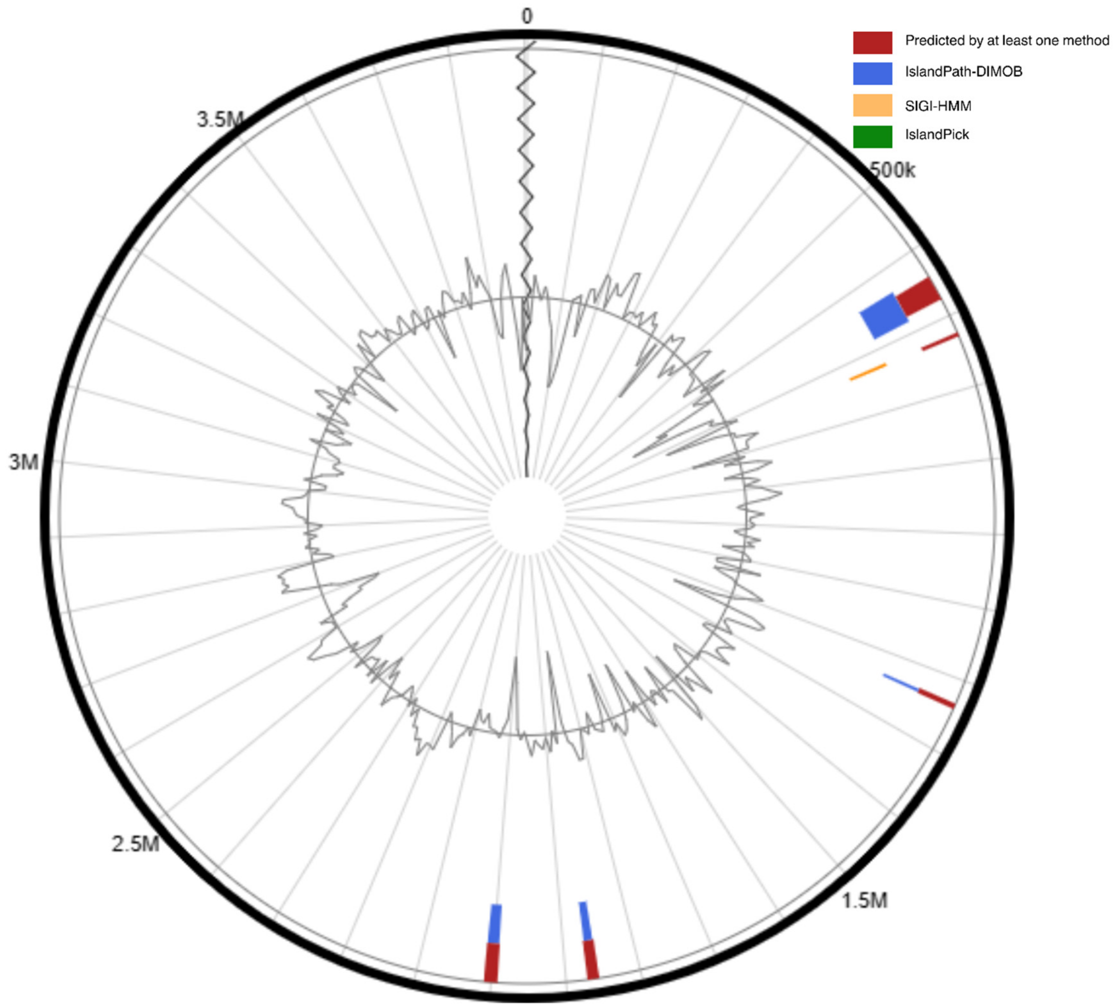

Supplementary Figure S2. Circular map generated by IslandViewer4 representing the locations of genomic islands within the genome of *Bacillus* sp. strain BACIII.

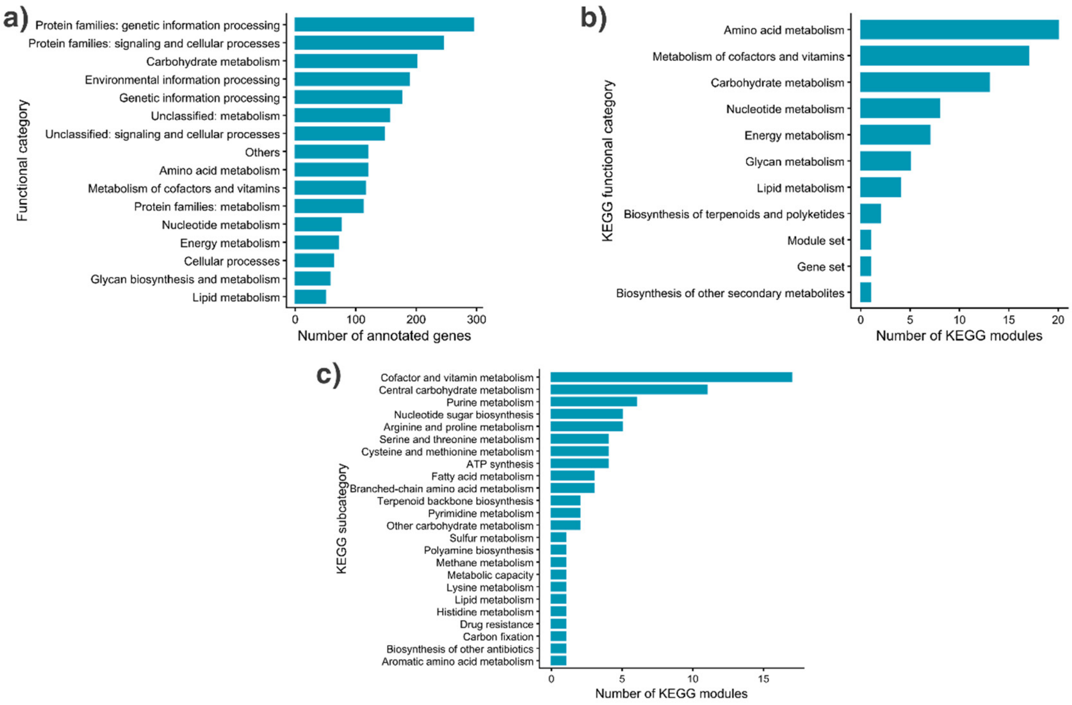

Supplementary Figure S3. Prediction of functional characteristics encoded in the *Bacillus* sp. BACIII genome. In a-c) functional annotation obtained with BlastKOALA. a) Distribution of annotated genes in the main functional categories of KEGG. b) Number of KEGG modules assigned to each functional category. c) Distribution of KEGG subcategories based on module annotation.

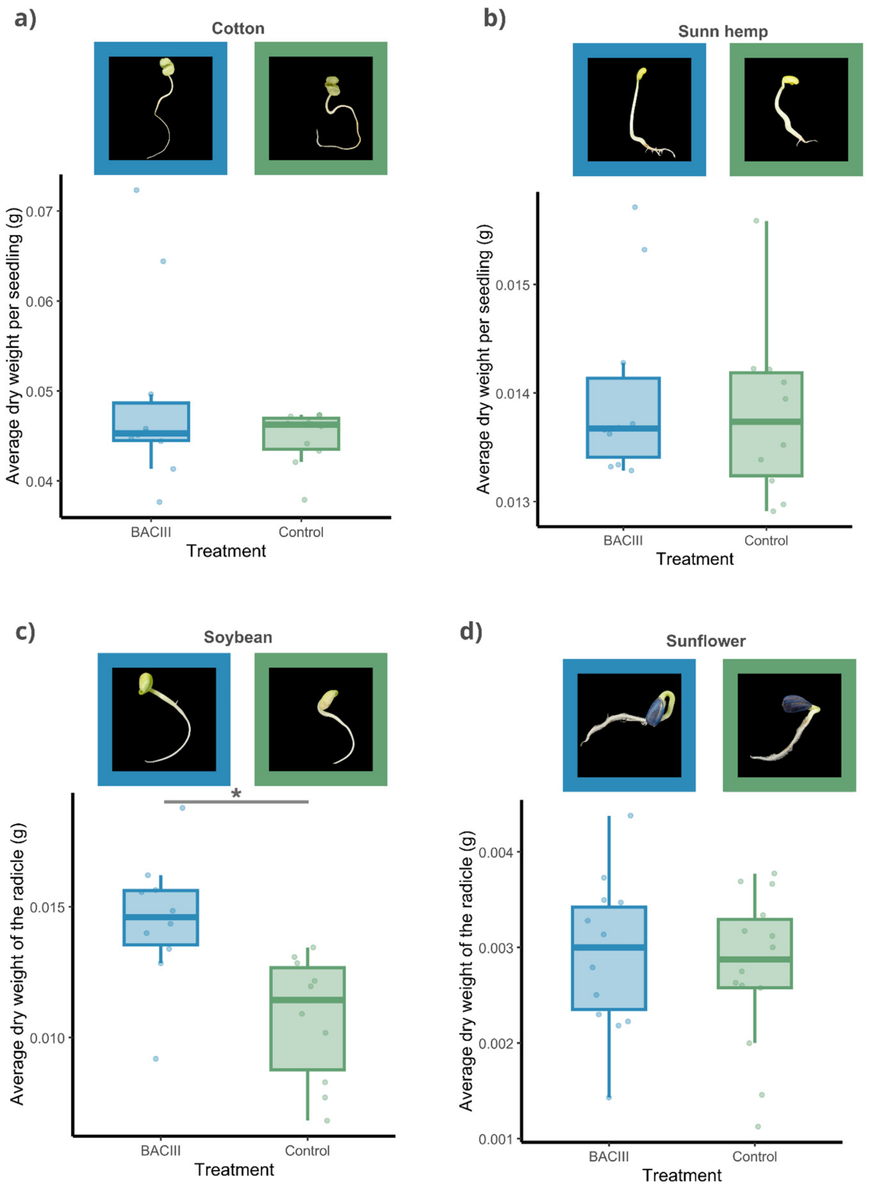

Supplementary Figure S4. Effect of *Bacillus* sp. strain BACIII treatment on seedling dry weight of four agricultural plant species. Panels show the average dry weight per seedling (a– cotton and b– sunn hemp) and the average dry weight of the radicle (c– soybean and d– sunflower) under two treatments: BACIII (bacterial inoculation) and Control (2% sucrose broth). Asterisks indicate statistically significant differences between treatments within the same species and parameter ($p \leq 0.05$), based on Student's t-test or Wilcoxon-Mann-Whitney test: "*" $p = 0.05$–0.02; "**" $p = 0.01$; "***" $p = 0.001$. Sample size per treatment: $n = 10$ for cotton; $n = 10$ for sunn hemp; $n = 5$ for soybean; $n = 14$ for sunflower.

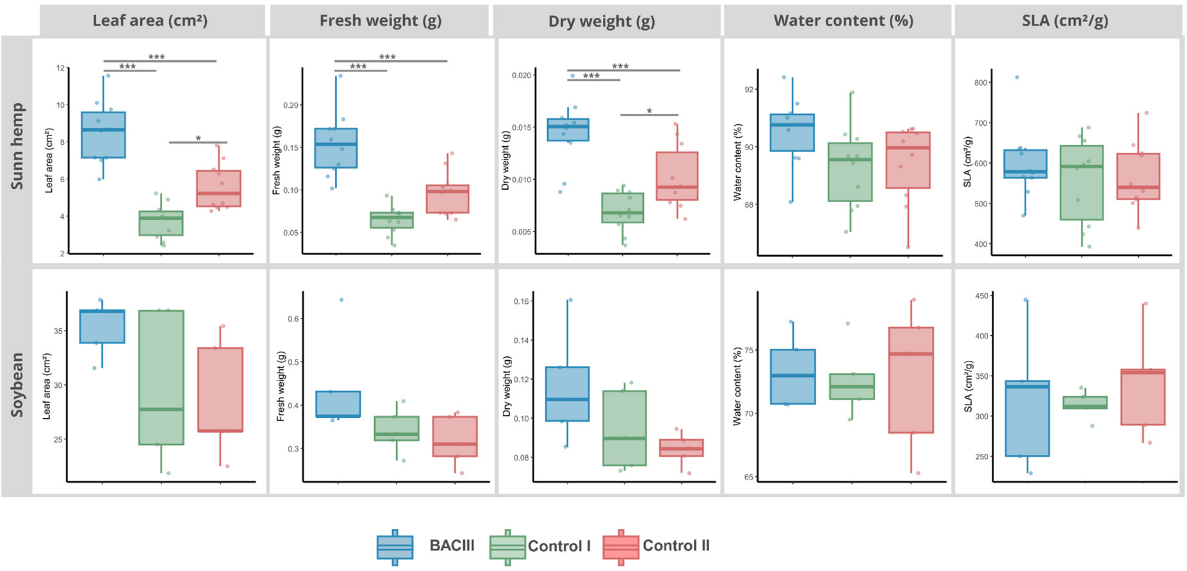

Supplementary Figure S5. Effect of *Bacillus* sp. strain BACIII treatment on leaf morphological traits of two plant species. Leaf area (cm^2), fresh and dry weight (g), water content (%), and specific leaf area (SLA, $cm^2 \cdot g^{-1}$) of sunn hemp (*Crotalaria juncea*, cv. C52333-C) and soybean (*Glycine max*, cv. Brasmax Olimpo IPRO 80I82RSF) under three treatments: BACIII (bacterial inoculation), Control I (water only), and Control II (culture medium without bacteria). Asterisks denote statistically significant differences between treatments within the same species and parameter ($p \leq 0.05$), based on ANOVA followed by Tukey's HSD test or Kruskal-Wallis followed by Dunn's multiple comparison test with Bonferroni correction:"*" p = 0.05–0.02; "**" p = 0.01; "***" p = 0.001. Sample size per treatment: n = 10 for sunn hemp; n = 5 for soybean.

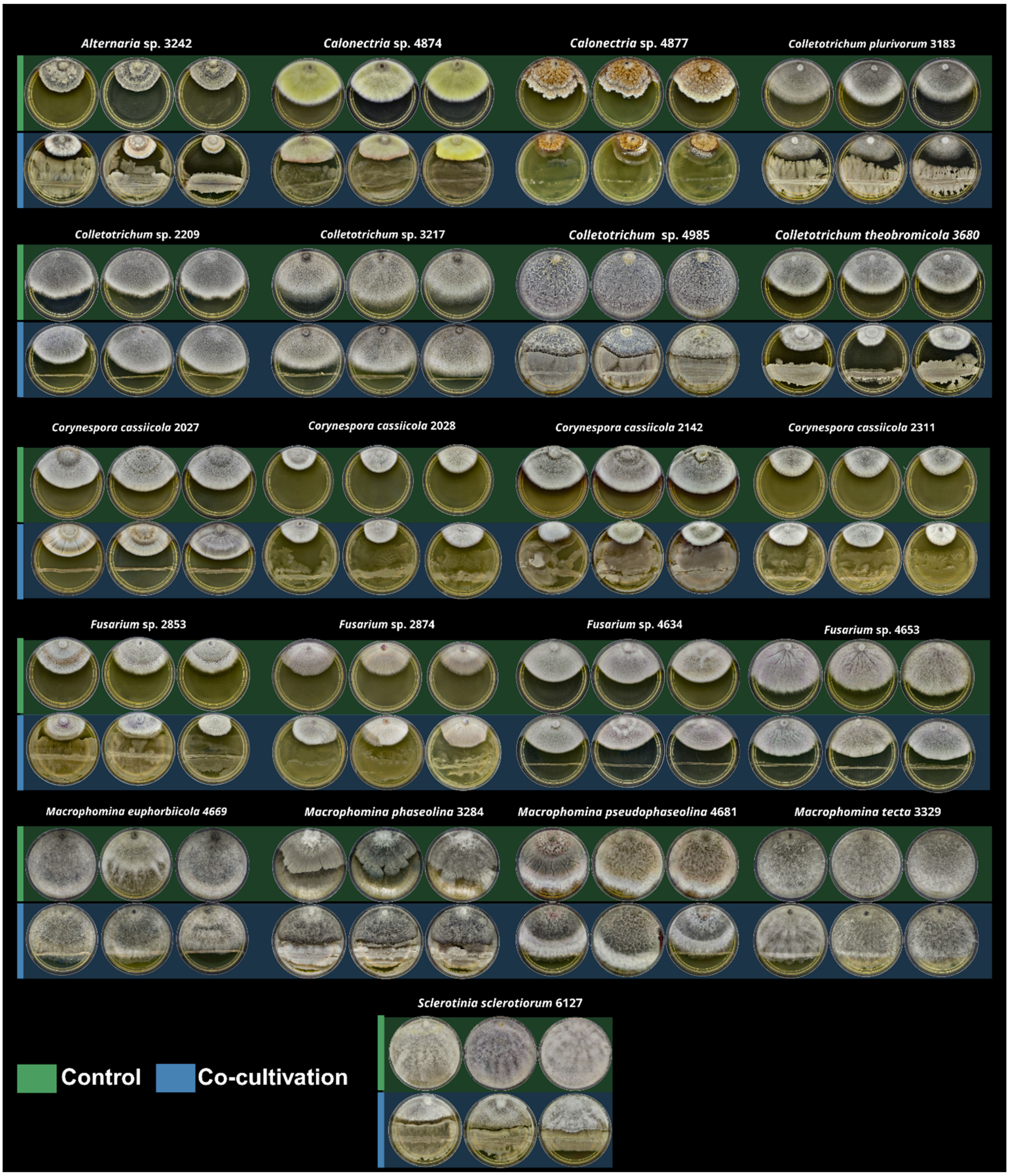

Supplementary Figure S6. Double culture assay of the *Bacillus* sp. BACIII strain against phytopathogenic fungi.

Supplementary Table S3. Results of statistical tests for the co-culture assay of the bacterial strain *Bacillus* sp. BACIII against different phytopathogenic fungi.

Genus	Specie	.y.	Group 1	Group 2	N1	N2	Statistic	DF	p	Test	Shapiro_ctrl	Shapiro_bac	Levene_p	p.adj	p.adj.signif
Alternaria	*Alternaria* sp. *3242*	Growth (mm)	BACIII	Control	10	10	-11,87550362	18	0,000000000597	t-test	0,609663374	0,949972759	0,3404932027	0,000000000597	****
Calonectria	*Calonectria* sp. *4874*	Growth (mm)	BACIII	Control	10	10	-15,08915272	18	0	t-test	0,7624948546	0,7480683588	0,5315931013	0	****
Calonectria	*Calonectria* sp. *4877*	Growth (mm)	BACIII	Control	10	10	11		0,0034	wilcox	0,001390148093	0,3178147119	0,891412934	0,0034	**
Colletotrichum	*C. plurivorum 3183*	Growth (mm)	BACIII	Control	10	10	-14,82063247	18	0	t-test	0,0953526759	0,5351673719	0,4399884286	0	****
Colletotrichum	*Colletotrichum* sp. *2209*	Growth (mm)	BACIII	Control	10	10	36		0,304	wilcox	0,006794147626	0,6146170454	0,3748280691	0,304	ns
Colletotrichum	*Colletotrichum* sp. *3217*	Growth (mm)	BACIII	Control	10	10	-4,254892301	18	0,000476	t-test	0,8904735097	0,1371132821	0,6581712624	0,000476	***
Colletotrichum	*Colletotrichum* sp. *4985*	Growth (mm)	BACIII	Control	10	10	0		0,000145	wilcox	0,001171076256	0,2466926481	0,1149539633	0,000145	***
Colletotrichum	*C. theobromicola 3680*	Growth (mm)	BACIII	Control	10	10	-12,12315445	18	0,000000000428	t-test	0,3194538732	0,7173662119	0,2940381096	0,000000000428	****
Corynespora	*C. cassiicola 2027*	Growth (mm)	BACIII	Control	10	10	27,5		0,0948	wilcox	0,668697761	0,01290114818	0,3177369684	0,0948	ns
Corynespora	*C. cassiicola 2028*	Growth (mm)	BACIII	Control	10	10	-3,275847762	18	0,0042	t-test	0,9476299665	0,4376182793	0,9315153143	0,0042	**
Corynespora	*C. cassiicola 2142*	Growth (mm)	BACIII	Control	10	10	-8,858315353	18	0,0000000557	t-test	0,6498517973	0,5847442954	0,5364375374	0,0000000557	****
Corynespora	*Corynespora cassiicola 2311*	Growth (mm)	BACIII	Control	10	10	-5,443996397	18	0,0000359	t-test	0,5315962176	0,7521018322	0,1647753205	0,0000359	****
Fusarium	*Fusarium* sp. *2853*	Growth (mm)	BACIII	Control	10	10	-7,817993908	18	0,00000034	t-test	0,6851907298	0,05100812738	0,475612513	0,00000034	****
Fusarium	*Fusarium* sp. *2874*	Growth (mm)	BACIII	Control	10	10	-5,947207096	18	0,0000126	t-test	0,872445156	0,08657152645	0,1916927618	0,0000126	****
Fusarium	*Fusarium* sp. *4634*	Growth (mm)	BACIII	Control	10	10	-8,907912635	18	0,0000000513	t-test	0,1415428076	0,5283918275	0,4407331369	0,0000000513	****
Fusarium	*Fusarium* sp. *4653*	Growth (mm)	BACIII	Control	10	10	-11,42249416	18	0,00000000111	t-test	0,1404226428	0,6289552947	0,4586897639	0,00000000111	****

Macrophomina	*M. euphorbiicola 4669*	Growth (mm)	BACIII	Control	10	10	22,5		0,0386	wilcox	0,03313764206	0,6762386628	0,1009683659	0,0386	*
Macrophomina	*M. phaseolina 3284*	Growth (mm)	BACIII	Control	10	10	0		0,000122	wilcox	0,0001995758122	0,1717573029	0,537821246	0,000122	***
Macrophomina	*M. pseudophaseolina 4681*	Growth (mm)	BACIII	Control	10	10	-0,5514881433	18	0,588	t-test	0,1045502524	0,1341905959	0,4762128707	0,588	ns
Macrophomina	*M. tecta 3329*	Growth (mm)	BACIII	Control	10	10	18,5		0,015	wilcox	0,0003868466429	0,523180342	0,007082028505	0,015	*
Sclerotinia	*S. sclerotiorum 6127*	Growth (mm)	BACIII	Control	10	10	0		0,000146	wilcox	0,0001122484505	0,2185337168	0,2914145743	0,000146	***

y: Observed value of the response variable;
Group1: First group compared in the statistical test (BACIII);
Group2: Second group compared in the statistical test (Control);
N1: Number of observations in Group1;
N2: Number of observations in Group2;
Statistic: Test statistic calculated for the comparison;
DF: Degrees of freedom associated with the test;
p: *p*-value obtained from the comparison before multiple-testing correction;
Test: Type of statistical test applied;
Shapiro_ctrl: *p*-value of the Shapiro–Wilk test for the control group;
Shapiro_bac: *p*-value of the Shapiro–Wilk test for the treated group;
Levene_p: *p*-value of Levene's test assessing variance homogeneity between groups;
p.adj: Adjusted *p*-value after multiple-comparison correction;
p.adj.signif: Categorical significance of the adjusted *p*-value (e.g., *, **, ***, ns). Asterisks indicate statistically significant differences between treatments within the same species and parameter ($p \leq 0.05$), based on Student's t-test or Wilcoxon–Mann–Whitney test: "*" $p = 0.05$–0.02; "**" $p = 0.01$; "***" $p = 0$.
